# Passive and Active Microrheology for Biomedical Systems

**DOI:** 10.3389/fbioe.2022.916354

**Published:** 2022-07-05

**Authors:** Yating Mao, Paige Nielsen, Jamel Ali

**Affiliations:** ^1^ Department of Chemical and Biomedical Engineering, FAMU-FSU College of Engineering, Tallahassee, FL, United States; ^2^ National High Magnetic Field Laboratory, Tallahassee, FL, United States

**Keywords:** microrheology, biomacromolecules, complex fluids, viscoelasticity, heterogeneity, mechanobiology

## Abstract

Microrheology encompasses a range of methods to measure the mechanical properties of soft materials. By characterizing the motion of embedded microscopic particles, microrheology extends the probing length scale and frequency range of conventional bulk rheology. Microrheology can be characterized into either passive or active methods based on the driving force exerted on probe particles. Tracer particles are driven by thermal energy in passive methods, applying minimal deformation to the assessed medium. In active techniques, particles are manipulated by an external force, most commonly produced through optical and magnetic fields. Small-scale rheology holds significant advantages over conventional bulk rheology, such as eliminating the need for large sample sizes, the ability to probe fragile materials non-destructively, and a wider probing frequency range. More importantly, some microrheological techniques can obtain spatiotemporal information of local microenvironments and accurately describe the heterogeneity of structurally complex fluids. Recently, there has been significant growth in using these minimally invasive techniques to investigate a wide range of biomedical systems both *in vitro* and *in vivo*. Here, we review the latest applications and advancements of microrheology in mammalian cells, tissues, and biofluids and discuss the current challenges and potential future advances on the horizon.

## Introduction

Rheology is the investigation of how materials deform in response to stress. Bulk rheology measures a material’s mechanical response to stress at a macroscopic scale, most often by employing a shear rheometer (Chen et al., 2010). Here, linear or oscillatory shear is applied to soft matter of interest confined within a geometry (e.g., parallel plates, cone and plate, *etc*.). Required sample volumes depend on the geometries used, however at the smallest scales typically tens of microliter volumes are required, which can limit the evaluation of biological samples that can be difficult to obtain and are available in only small (<10 µl) quantities. Rheometer geometries that size from 20 to 25 mm in diameter require sample volumes of ∼ 10^1^–102 μl and can be used on soft materials like hydrogels (Bonino et al., 2011; Zuidema et al., 2014). Smaller geometries (6–8 mm) use sample sizes as small as a few microliters and can be used for biomaterials with high mechanical properties such as cartilage (Perni and Prokopovich, 2020). Microscale methods minimize the sample size. Microrheology employs micro/nanoparticles as mechanical probes and can target and interrogate the mechanical properties of specific tissues and cell types (Waigh, 2005; Weihs et al., 2006). Small scale rheological techniques can also extend the frequency range of conventional rheology by orders of magnitude (Table 1). Microrheology encompasses a collection of techniques that fall into two broad categories, active and passive, defined by the force exerted on probe particles (Furst and Squires, 2017). Particles passively driven by thermal energy fluctuations undergo translational and rotational Brownian motion that can be measured and used to determine structural and rheological properties (Squires and Mason, 2010). Common passive techniques include dynamic light scattering (DLS) (Stetefeld et al., 2016; Cai et al., 2021), diffusing wave spectroscopy (DWS) (Pine et al., 1988; Pine et al., 1990; Weitz et al., 1993), and video particle tracking (VPT). When using an active method, probe particles are manipulated by an external force, typically generated by magnetic or optical fields, produced by magnetic tweezers (MT) (Bausch et al., 1999; Pankhurst et al., 2003) and optical tweezers (OT) (Yao et al., 2009; Norregaard et al., 2017). The motion of particles suspended in a medium of interest provides dynamic information about the viscous and elastic properties of the surrounding environment and can be used to determine microstructural information.

**TABLE 1:** Summary of benefits, limitations, and operating regimes of microrheological techniques, including dynamic light scattering (DLS), diffusing wave spectroscopy (DWS), video particle tracking (VPT), optical tweezers (OT), magnetic tweezers (MT).

Technique	Benefits	Limitations	Frequency range (Hz)	Shear modulus (Pa)	Ref
DLS	Commonly available equipment	Cannot resolve spatial heterogeneity. Requires samples allowing over 90% of light transmission	10^−1^ – 10^6^	10^−3^ – 10^4^	Krajina et al. (2017)
DWS	Allows probing opaque samples; can access a broad range of frequencies	Cannot resolve spatial heterogeneity	10^0^–10^7^	10^−1^ – 10^4^	Mason et al. (1997b)
					Huh and Furst, (2006)
VPT	Allows MPT and TPM for an average measurement and SPT analysis to investigate heterogeneity	Limited to soft materials, not applicable to stiff materials and non-equilibrium systems	10^0^–10^2^	10^−5^ – 10^0^	Furst and Squires, (2017)
					Li et al. (2020)
OT	Allows passive and active measurements; can be used to manipulate objects in the range of 10 μm–100 nm	Usually requires stiffness calibration of laser traps	10^−1^ – 10^4^	10^−3^ – 10^2^	Preece et al. (2011)
					Taylor et al. (2013)
MT	Produces biologically safe magnetic force fields and field gradients to manipulate probe particles	Requires the use of magnetic probes	10^−2^ – 10^3^	10^−3^ – 10^4^	Evans et al. (2009)
					Fabry et al. (2001)

The theoretical prediction of Brownian motion was derived by Albert Einstein in 1905 (Einstein, 1905) and quantitatively confirmed by Jean Perrin in 1908 (Haw, 2002). Freundlich and Seifriz performed the first microrheological measurement in 1922 (Freundlich and Seifriz, 1923), using ion-filled gelatin driven by magnetic field gradients. Decades later, active microrheology incorporating magnetic forces started to grow as well-characterized magnetic probes were synthesized in the 1990s. The development of passive microrheology was facilitated by the advancement of optical and laser technology, with passive measurements obtained in DLS and DWS experiments (Pusey and Van Megen, 1989; Schepper et al., 1989). In seminal work, Mason and Weitz used the generalized Stokes-Einstein relation (GSER) to analyze fluid viscoelasticity using passively diffusing particles, and today their analytical estimation of the shear moduli has become a standard tool in microrheology (Mason et al., 1997a; Mason, 2000). Microrheology extends the range of probing forces and frequencies of conventional rheometry and enables the acquisition of spatiotemporal data to characterize heterogeneous materials. These benefits make microrheology ideally suited for analyzing biological samples that are often complex, heterogeneous, and inherently non-equilibrium systems. Researchers have taken these advantages of microrheology to examine biological systems in many contexts, including organelle transport in live cells, cell softening and extracellular environment stiffening during malignant tumor progression, and modeling drug penetration through the mucus of diseased cells.

In this review, a brief introduction of common passive and active microrheology techniques and their methodologies is first presented, starting with passive methods as their experimental setup and data acquisition are often simple and straightforward. Following the passive methods are the active techniques that require specific instrumentation while providing a wider range of measurement. The second section reviews the latest microrheological applications and advancements in mammalian cells, tissues, and biofluids that are categorized by organ systems. Lastly, we discuss the limitations of microrheological applications in assessing biological systems and provide a perspective for the future of small-scale biomedical rheology.

## Microrheological Methodology

### Passive Microrheology

Common passive microrheology techniques include VPT, DLS, and DWS. In passive methods, freely diffusing particles exhibit Brownian motion that is driven by thermal energy. The mobility of these particles is used to obtain the microscopic viscoelasticity and structure of the local fluid medium. More specifically, particle trajectories or scattered light intensities are analyzed to compute single-particle or ensemble-averaged mean-squared displacement (MSD), which can be quantitatively interpreted into viscosity and shear modulus of the microenvironment. Since the Brownian motion of probe particles is driven by thermal energy (*k*_B_ *T*), passive measurements usually fall within the linear viscoelastic regime. Therefore, passive methods are advantageous for characterizing soft materials. However, passive techniques are also limited to relatively soft media in which probe particles display discernable displacement over time; otherwise, the probe particles are observed to be confined in space and time, corresponding to the material response of a purely elastic solid.

**FIGURE 1:**
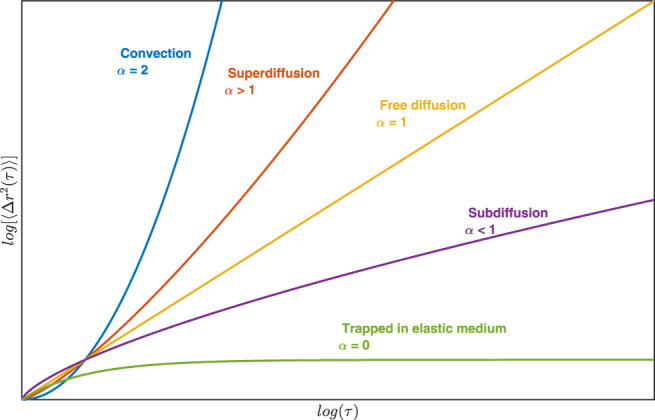
Plot of MSD as a function of time indicating different types of particle motion in the medium.

In Newtonian fluids that are purely viscous, probe particles are diffuse freely, displaying thermally driven Brownian motion. The ensemble-averaged MSD, $\langle \Delta r^{2}\left(\tau \right)\rangle$ , of probe particles linearly increases with time as described by:

$$\langle \Delta r^{2}\left(\tau \right)\rangle =2dD_{t}\tau$$ (1)

where 〈⋅〉 indicates ensemble average, d is the dimensionality of measurement (d=1, 2, 3) , $D_{t}$ is the translational diffusion coefficient, and τ is the lag time. For a spherical particle in a Newtonian fluid with a radius of a , its $D_{t}$ is related to the viscosity ( η ) of fluid by Stokes-Einstein relation (Einstein, 1905):

$$D_{t}=\;\frac{k_{B}T}{6\mathrm{\pi \eta a}}$$ (2)

where $k_{B}$ is the Boltzmann constant and T is the temperature of the medium. The MSD of probe particles in viscoelastic materials is estimated to follow a power law relation empirically (Mason, 2000):

$$\langle \Delta r^{2}\left(\tau \right)\rangle =\langle \Delta r^{2}\left(\frac{1}{\omega }\right)\rangle {\left[\mathrm{\omega t}\right]}^{\alpha \left(\omega \right)}$$ (3)

where $\langle \Delta r^{2}\left(\frac{1}{\omega }\right)\rangle$ is the magnitude of $\Delta r^{2}\left(\tau \right)$ at $\tau =\frac{1}{\omega }$ , and α is the power law exponent ( 0≤α≤1 ), which is also the logarithmic slope of MSD versus τ . Freely diffusing probe particles in Newtonian fluids show α=1 (Eq. 1). Probe particles in subdiffusive motion in viscoelastic fluids show 0<α<1 , and α=0  indicates trapped motion of particles as in a purely elastic material (Hookean solid), as shown in Figure 1.

The frequency-dependent complex modulus can be related to the Fourier transform of the MSD by the generalized Stokes-Einstein relation (GSER) (Mason, 2000), yielding the algebraic estimate of the complex modulus:

$$G^{*}\left(\omega \right)\approx \frac{dk_{B}T}{3\mathrm{\pi a}\langle \Delta r^{2}\left(\frac{1}{\omega }\right)\rangle \Gamma \left[1+\alpha \left(\omega \right)\right]}$$ (4)

where Γ is the gamma function. The complex modulus can be decomposed into the shear elastic (storage) and viscous (loss) moduli using Euler’s equation:

$$G^{\prime }\left(\omega \right)=|G^{*}\left(\omega \right)|\cos\frac{\pi \alpha \left(\omega \right)}{2}$$ (5)

$$G^{\prime \prime }\left(\omega \right)=|G^{*}\left(\omega \right)|\sin\frac{\pi \alpha \left(\omega \right)}{2}$$ (6)

Shear moduli can also be solved in the Laplace domain (Mason et al., 1997a; Mason et al., 1997b; Xu et al., 1998; Winter and Mours, 2006). Li et al. compared the accuracy of results determined by different approaches (Li et al., 2020).

The GSER is valid for conditions where probe particles are surrounded by a continuous, spatially homogeneous, isotropic, incompressible media that is at thermal equilibrium and displays linear response. In addition, the relation assumes fluid inertia is negligible, probe particles move at equilibrium or quasi-equilibrium state with a no-slip boundary surface condition, and that there are no disturbances from the far field (Mason and Weitz, 1995; Mason et al., 1997a). However, there are cases where these assumptions are violated, which will be discussed later in this review.

Within soft matter, thermal fluctuation not only induce translation of probe particles, but also rotational motion. The ensemble-average mean squared angular displacement, $\langle \Delta^{2}\theta \left(\tau \right)\rangle$ , in one dimension of the spherical coordinate system, is related to rotational diffusion coefficient, $D_{r}$ , of a spherical particle by (Cheng and Mason, 2003; McNaughton et al., 2011):

$$\langle \Delta \theta^{2}\left(\tau \right)\rangle =2D_{r}\tau$$ (7)

In a Newtonian fluid, the viscosity can also be determined by the rotational diffusion coefficient using the Stokes-Einstein relation, such that:

$$D_{r}=\frac{k_{B}T}{8\pi \eta a^{3}}$$ (8)

The shear modulus is given by the GSER in the Laplace domain (Cheng and Mason, 2003), such that:

$$\tilde{G}\left(s\right)=s\tilde{\eta }\left(s\right)=\frac{k_{B}T}{4\pi a^{3}s\langle \Delta {\tilde{\theta }}^{2}\left(s\right)\rangle }$$ (9)

The inverse Laplace transformation into the time domain yields the creep compliance (Cheng and Mason, 2003):

$$J\left(t\right)=\frac{4\pi a^{3}\langle \Delta \theta^{2}\left(t\right)\rangle }{k_{B}T}$$ (10)

#### Dynamic Light Scattering

**FIGURE 2:**
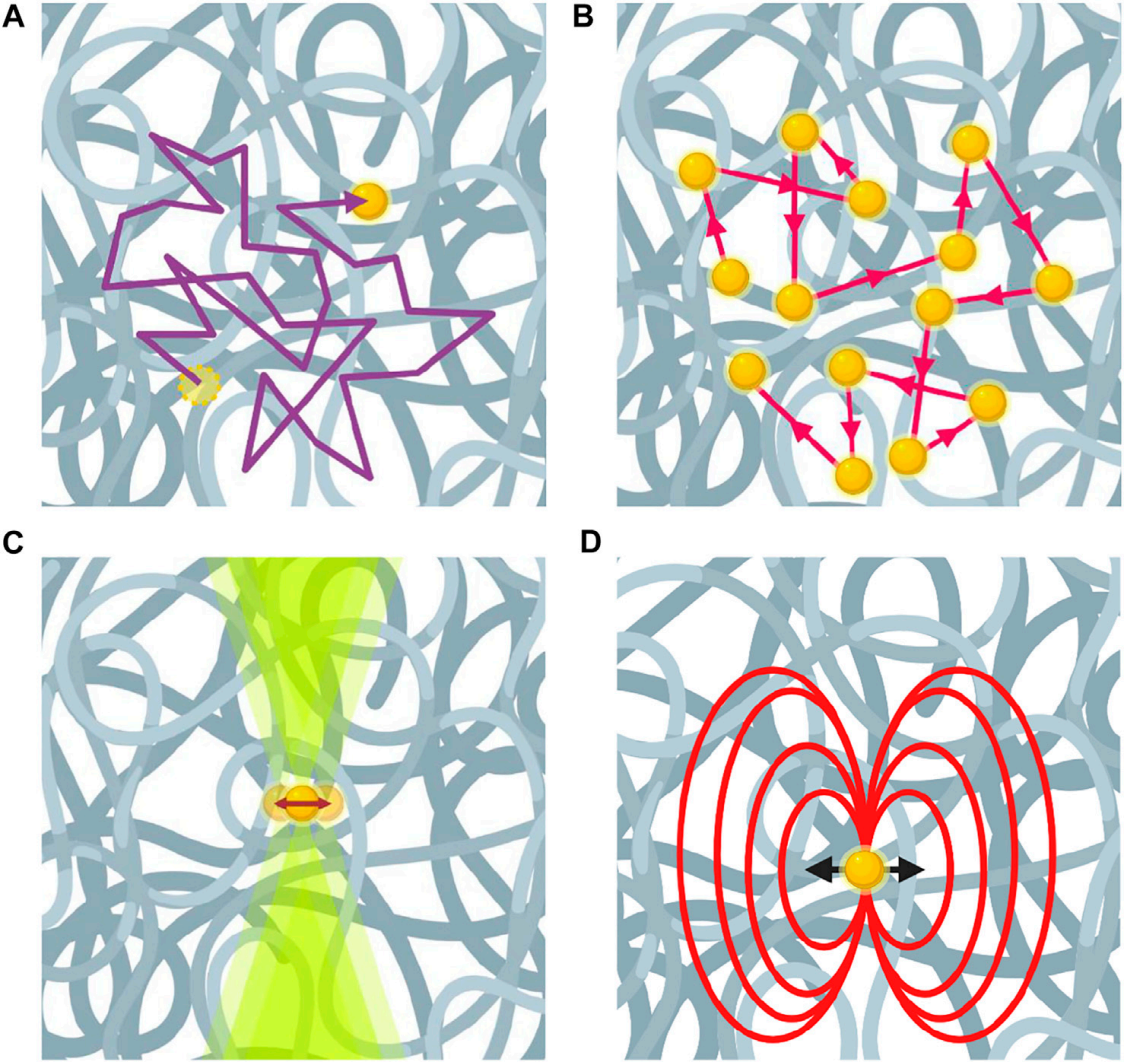
Microrheology using different techniques for passive and active measurements. **(A)** Video particle tracking technique visually tracks the trajectories of probe particles. **(B)** Multiple-scattered laser in diffusing wave spectroscopy experiment reflect Brownian motion of probe particles. **(C)** Optical tweezers measure the force and displacement on a probe particle. **(D)** Magnetic field exerted on a probe particle by magnetic tweezers.

Dynamic Light Scattering (DLS) forms the foundation of passive microrheology as much of today’s microrheology theoretical framework has arisen from light scattering research (Tanaka et al., 1973; Mason and Weitz, 1995). DLS measures the variation of scattered light intensity of probe particle as a function of time—yielding an autocorrelation function, using coherent monochromatic light source and detection optics. Changes in scattered light intensity are due to the thermally driven motion of embedded colloids (Figure 2B). The field correlation function $g_{1}$ is related to the intensity correlation function $g_{2}$ by the Siegert relation (Van Megen and Pusey, 1991; Gardel et al., 2005; Furst and Squires, 2017):

$$g_{2}\left(t\right)=\beta {\left|g_{1}\left(t\right)\right|}^{2}+1=\frac{\langle I\left(t_{0}\right)I\left(t_{0}+t\right)\rangle }{I^{2}}$$ (11)

Where ***I*** is the light intensity and 〈·〉 indicates its average, β is the coherence factor, which depends on the experimental setup. DLS experiments are performed with the assumption that only single scattering events occur. The detector (i.e. photodiode) used to measure scattered light can be positioned at a range of detection angles, θ , corresponding to the scattering wave vector, q :

$$q=\frac{4\mathrm{n\pi}}{\lambda }\sin\left(\frac{\theta }{2}\right)$$ (12)

where n is the refractive index of the medium, and λ is the wavelength of the incident laser beam in the medium. For scattering in a Newtonian fluid, the field correlation function follows a single exponential decay. The field correlation function, $g_{1}\left(\tau \right)$ , measured at the wave vector, $g_{1}\left(\tau ,q\right)$ , is related to the diffusion coefficient of probe scatterers by:

$$g_{1}\left(\tau ,q\right)=\exp\left(-Dq^{2}\tau \right)$$ (13)

Substituting D with Eq. 1, the MSD can be obtained from the field correlation function:

$$g_{1}\left(\tau ,q\right)=\exp\left[-\frac{q^{2}\langle \Delta r^{2}\left(\tau \right)\rangle }{2d}\right]$$ (14)

The MSD obtained from Eq. 14 can then be used to determine the frequency-dependent shear modulus by applying Eq. 4. DLS can be used for measurements at high frequencies (e.g. 10^3^ > Hz) and can be performed on polymer solutions without the addition of tracer particles (Tanaka et al., 1973). However, DLS is also limited to transparent samples, requiring over 90% light transmission, in order to ensure single scattering detection.

#### Diffusing Wave Spectroscopy

The limitations of DLS led to the development of diffusing wave spectroscopy (DWS). In contrast to DLS, light in DWS measurement needs to be highly scattered. Therefore, the slight movement of probes can cause a substantial change of the overall diffusion path, which extended light scattering to opaque systems. The experimental setup of DWS is similar to that of DLS. Two commonly used modes in DWS experiments are transmission and backscattering (Pine et al., 1990; Fahimi et al., 2017). In the high multiple photon scattering regime, the details of individual single scattering events are no longer relevant. Instead, the photon diffusion process can be described by the transport mean free path, $l^{*}$ , which is the average distance a photon travels in the sample fluid before propagation is randomized. Therefore, $l^{*}$ is the approximated step size of the random walk of photons. In this case of multiple-scattering, the correlation function is not angular-dependent and is determined by the contributions of all path lengths, s, weighted by their distribution P(s) , such that (Pine et al., 1990; Weitz et al., 1993):

$$g_{1}\left(\tau \right)=\int_{0}^{\infty }P\left(s\right)\;\exp\left[-\frac{k_{0}^{2}}{3}\langle \Delta r^{2}\left(\tau \right)\rangle \frac{s}{l^{*}}\right]\mathrm{ds}$$ (15)

where $k_{0}=\frac{2n\pi }{\lambda }$ is the magnitude of the wave vector, and $\frac{s}{l^{*}}$ reflects the number of steps in the corresponding path. P(s) can be determined using the diffusion model, considering the experiment geometry (transmission and backscattering). Then, $\langle \Delta r^{2}\left(\tau \right)\rangle$ can be determined numerically by inverting the field correlation function shown in Eq. 15. It is important to note that both DLS and DWS provide bulk average measurements, unlike other microrheological methods subsequently discussed (Cicuta and Donald, 2007).

#### Video Particle Tracking

In video particle tracking experiments, image sequences of moving probe particles in a sample fluid are recorded by a digital camera and subsequently tracked by linking particle positions between frames (Figure 2A). By using tracking algorithms, the MSD of the particles can be computed from their trajectories, extracted from the recorded series of images, allowing for the computation of viscoelastic properties of the sample fluid (Mason et al., 1997a). Taking advantage of the independent movement of probe particles in all dimensions within an isotropic system, 2D particle tracking is usually performed in the x-, y-plane. Moreover, fluorescence microscopy is more commonly used than bright field microscopy, because fluorescence microscopy generates images with bright features on a dark background that can typically resolve smaller probes (∼0.1 μm) than bright field microscopy methods, which also facilitates the use of many tracking algorithms that operate by determining the maximum brightness of an object in the field of view to locate centroid. Fluorescent labeling also allows probe particles to be easily located within tissues or cells. A five-step tracking algorithm originally developed by Cocker and Grier in IDL has been widely used in VPT analysis (Crocker and Grier, 1996; Crocker and Weeks, 2011). These routines have been adapted for MATLAB and Python (Daniel Blair, 2008; Crocker and Grier, 2016).

After obtaining the trajectories of probe particles, the ensemble-averaged MSD can be determined from the particle positions in each frame. Applying the ensemble-averaged MSD to Eq. 1 for Newtonian fluids and Eq. 3 for viscoelastic materials and solving for Eq. 2 (Newtonian) and (4) (viscoelastic), one obtains an average measurement of the medium. This analysis is termed multiple particle tracking (MPT).

VPT precision can be improved by quantifying and correcting the static and dynamic error of the particle tracking experiments (Savin and Doyle, 2005a). The static error is the intrinsic resolution of the system that can be measured by the fixed beads method. The dynamic error is attributed to the image acquisition mechanism of the camera, because particle motions are not recorded when the shutter of the camera opens to gain exposure. Therefore, for measurement with lag times longer than the shutter time, the camera-captured position of a particle is considered its average position during the shutter time (Savin and Doyle, 2005a; Savin and Doyle, 2005b).

#### General Considerations for Passive Microrheology

Some limitations of passive microrheology arise from the assumptions made in solving the Stokes and Einstein components of the GSER. Microstructure and heterogeneity are unavoidable in soft materials such as concentrated polymer solutions and the cytoplasm of cells. In the case of a structured material, when the probes are much smaller than the mesh size, they would diffuse freely and show measurement close to the interstitial fluid. If the probe is large enough that the surrounding can be treated as a continuum but still display Brownian motion, one would obtain consistent results as bulk measurements. Both results provide information about the environment at their length scale that should be interpreted properly (Squires and Mason, 2010). To achieve consistent microrheologial measurement with the bulk, approaches include comparing micro- and macrorheology measurements in their overlapping frequencies, test with a series of different size of probe particles and test with probes with different surface chemistries (McGrath et al., 2000; Lu and Solomon, 2002), and use two-point microrheology (TPM). TPM has been developed to reduce particle–environment interaction effects, such as the effect of particle surface chemistry and environmental heterogeneity, by correlating the motion two well-separated probe particles for measurement (Crocker et al., 2000; Crocker and Hoffman, 2007). Single particle tracking analysis (SPT) takes the time-averaged MSD of individual particles. As a result, the thermal fluctuation of each particle reflect the local environment surrounding each particle (Qian et al., 1991). The displacement of particles in a homogeneous environment should display a Gaussian distribution, and vice versa. Statistical analysis of the van Hove correlation function of particle displacement has been used to determine particle populations, thus heterogeneity of the material (Valentine et al., 2001; Dasgupta and Weitz, 2005).

At high frequencies (∼10^7^ Hz for 1 μm particles), fluid inertia can impact particle motion (Mason and Weitz, 1995; Mason, 2000; van Zanten and Rufener, 2000). This inertial frequency is within the frequency range of DWS. VPT typically operates in the lower frequency range, fluid inertia remains negligible. But some latest models of high-speed camera can reach millions of hertz. In the case of exceeding the inertial frequency, the force on a particle should account for fluid inertia contribution and correct Eq. 4 accordingly (Indei et al., 2012; Schieber et al., 2013; Domínguez-García et al., 2014).

The surface chemistry of probe particles may induce strong interaction between probe particles and polymer filaments of the sample, which may affect the interpretation of microrheological measurements (Valentine et al., 2004). Undesired particle attachment to materials can be reduced by surface functionalization or coating (He and Tang, 2011), polyethylene glycol (PEG) surface fuctionalization is commonly used to achieve this goal (Valentine et al., 2004). Carboxylated particles have been shown to strongly adsorb to protein and used to probe the persistence length of fibrin filaments (Jahnel et al., 2008).

The conditions that cause GSER components to break down are discussed in the review by Squires et al. (Wu et al., 2020). GSER corrections considering cases such as compressible media, probe inertia, and slipping boundaries are discussed by Furst et al. (Furst and Squires, 2017).

The collective movement and vibration of probe particles can be reduced by performing two TPM analyses (Crocker and Hoffman, 2007; Corrigan and Donald, 2010). The collective motion of particle pairs has been used to characterize sample drift (Mason et al., 1996). Image process algorithms have been used to determine sample drift to reduce it and enabled particle tracking in living mice (Herráez-Aguilar et al., 2020; Wu et al., 2020).

Passive microrheology has been particularly useful to explore evolving and aging materials with minimal perturbation, such as polymer sol-gel transition and degradation (Gomez-Solano et al., 2013; Xing et al., 2018; McGlynn et al., 2020). Gelation should be slow enough to satisfy the GSER assumption that the material is at quasi-equilibrium (Furst and Squires, 2017). Time-cure superposition has been applied in microrheology to obtain master curves of the gelling material and characterize the gel point (Larsen et al., 2008; Larsen and Furst, 2008; Schultz and Furst, 2012; Wehrman et al., 2017) and predict polymer degradation (Larsen et al., 2008; Schultz et al., 2012). The crossover point of G′ and G″ has also been traditionally used to identify the gel point of materials. For a material exhibit a power law behavior such that $G^{\prime }\left(\omega \right)\propto \omega^{\alpha }$ and $G^{\prime \prime }\left(\omega \right)\propto \omega^{\alpha }$ , the Rouse dynamic model of α=0.5 has been used to identify the gel point that $G^{\prime }\left(\omega \right)=G^{\prime \prime }\left(\omega \right)$ (Savin and Doyle, 2007; Wehrman et al., 2017). For a probe particle that follows Eq. 4, this scaling can be converted to $\langle \Delta r^{2}\left(\tau \right)\rangle ∼\tau^{0.5}$ . Transient microrheology of gelling materials has been performed to identify the critical point of α=0.5 (Savin and Doyle, 2007; Xing et al., 2018).

Passive microrheology is limited to equilibrium systems that display linear responses. A material’s nonlinear behavior cannot be measured by passive methods (Squires and Brady, 2005; Furst and Squires, 2017; Zia, 2018). The driving force of passive microrheology is the thermal energy of the probe particles that allows non-invasive probing but also limits passive measurements to soft materials (Table 1). Nonequilibrium systems and materials with relatively higher mechanical properties can be evaluated by active approaches (Rich et al., 2011; Zia, 2018).

### Active Microrheology

Passive and active methods produce similar results in equilibrium systems. When using an active technique, probe particles are manipulated by an external force, commonly a magnetic or optical force. Therefore, active methods are more suitable for stiffer media with higher viscoelasticity. Active microrheology can also be used to study the heterogeneity for receiving a localized response from probes. Oscillatory and creep manipulations are usually performed in active operations. In creep experiments, pulses of a constant stress or shear rate are applied to measure how the sample recovers to its original state. Creep experiments are typically seen in both MT and OT manipulations. In oscillatory experiments, continuous sinusoidal shear stress or shear rate is applied on the probe particles to obtain the viscoelasticity from the phase shift of sample recovery. Oscillatory operation is commonly performed using OT; however, this is more challenging to accomplish using MT.

#### Magnetic Tweezers

MT combines magnetic manipulation of probe particles and video microscopy to track the motion of these particles and extract their viscoelastic properties. Strong magnets or electromagnetic coils are commonly used to generate magnetic fields in MT experiments. A constant magnetic field exerts a torque on magnetic particles that causes them to rotate. A more complex experimental setup is required for tracking the rotational motion of a spherical particle. The gradient of a magnetic field generates a force on the magnetic particle that induces translational motion (Figure 2D). MT can be designed in different configurations to meet the need of experiments (Kriegel et al., 2017). Commonly used probe particles in MT experiments have paramagnetic, ferromagnetic, and superparamagnetic bodies. Paramagnetic and superparamagnetic particles need an external magnetic field to maintain dipole moment, while ferromagnetic particles retain dipole moment without an external field. Moreover, superparamagnetic particles experience smaller torques than ferromagnetic particles. The dipole moments are rapidly adjusted on superparamagnetic particles, avoiding rotational motion; therefore, they are typically used for measuring translational motion.

For a particle with a dipole moment ( m ) induced by a magnetic field ( B ), the magnetic force is given by (Wilson and Poon, 2011):

$$F=\nabla \left(\bar{m}\cdot \bar{B}\right)=\frac{4\pi a^{3}}{\mu_{0}}\left(\frac{\mu_{r}-1}{\mu_{r}+2}\right)\left(\bar{B}\cdot \right)\nabla \bar{B}$$ (16)

where $\mu_{r}$ is the particle’s relative permeability. There are three methods commonly used for microrheological measurements: constant force, creep-response, and oscillatory. The constant force method is typically used to measure the viscosity of a Newtonian fluid by balancing the drag force, $F_{m}=6\pi \eta av$ , of a probe particle with a constant magnetic force, where v is the velocity of a particle. When both the magnetic field and dipole moments are unknown, the force exerted on a particle can be calibrated in a fluid with a known viscosity of η using this method (Furst and Squires, 2017). In the creep-response mode, a rectangular force pulse, $F_{m}\left(t\right)$ , induces displacement, x(t) , on a probe particle in the direction of the force. The creep compliance, J(t) , of the sample can be determined by:

$$J\left(t\right)=\frac{6\mathrm{\pi ax}\left(t\right)}{F_{m}\left(t\right)}$$ (17)

J(t) can be further interpreted as the shear elastic and storage moduli (Bausch et al., 1998; Uhde et al., 2005). In the oscillatory method, oscillatory force is applied to the probe particles at a controlled amplitude and frequency, such that $F_{m}\left(t\right)=F_{0}\text{exp}\left(i\omega t\right)$ . The displacement of the particles can be expressed in the same form with a phase shift, $x\left(t\right)=x_{0}\text{exp}\left[i\left(\omega t-\varphi \right)\right]$ . Shear elastic and viscous moduli are given by (Furst and Squires, 2017; Joyner et al., 2020):

$$G^{\prime }\left(\omega \right)=\frac{F_{0}}{6\mathrm{\pi a}|x_{0}\omega |}\mathrm{cos \varphi}\left(\omega \right)$$ (18)

$$G^{\prime \prime }\left(\omega \right)=\frac{F_{0}}{6\mathrm{\pi a}|x_{0}\omega |}\mathrm{sin \varphi}\left(\omega \right)$$ (19)

Microrheological studies using MT have also been performed by inducing rotational motion on monodispersed beads (Fabry et al., 2001), chains of beads (Wilhelm et al., 2003a; Wilhelm et al., 2003b), and other anisotropic probes such as wires, rods, and disks (Zhang et al., 2011; Chevry et al., 2013).

#### Optical Tweezers

OT uses a highly focused laser beam to manipulate single dielectric probe particles (Figure 2C). This technique can probe samples both passively and actively. Using an OT system that employs conventional back-focal-plan interferometry, the first step of OT experiments is to calibrate trap stiffness, which is usually performed using a particle with known size in a fluid with known viscosity. Particles in the optical trap experience a scattering force generated by the radiation pressure and a gradient force generated by the gradient of field intensity. The scattering force is usually balanced out by gravitational force, and the gradient force forms the optical trap. In OT systems with improved back-focal-plane interferometry, where the force sensor detects light momentum change instead of absolute light momentum, *in situ* calibration is no longer required (Smith et al., 2003; Farré and Montes-Usategui, 2010).

In OT experiments, a dielectric probe particle with a refractive index of n_2_ is trapped at the focus of the laser beam in a medium with a refractive index of n_1_. Manipulation of the particle is achieved by changing the position of the laser focus. The off-focus force on a probe particle is determined by (Gardel et al., 2005):

$$F_{\mathrm{ot}}=-\frac{\alpha n_{1}V}{cR^{2}}\left(\frac{n_{2}^{2}-n_{1}^{2}}{n_{2}^{2}+2n_{1}^{2}}\right)I_{0}e^{-\frac{r^{2}}{R^{2}}}re_{r}=-k_{\mathrm{ot}}re^{-\frac{r^{2}}{R^{2}}}e_{r}$$ (20)

where α is a geometrical factor of order one, c is the speed of light, V is the volume of particle, R is the radius of the waist of the laser beam, r is the distance from the trap center, $I_{0}$ is the intensity of the incident light, $e_{r}$ is the unit vector from the beam center, and $k_{ot}=2\pi \gamma f_{c}$ is the spring constant of the optical trap, γ is the drag coefficient, and $f_{c}$ is the cornering frequency of the power spectrum. The relation given by Eq. 20 follows Hooke’s law for small displacements ( r≪R ), and $k_{ot}$ is considered constant. The optical force can be determined from the displacement of particles, the escape force required to move the particle out of the trap, or the Brownian motion of the trapped particle.

Knowing the oscillation amplitude $A_{L}$ of the laser beam, the amplitude *A* of the displacement as a function of oscillation frequency, *ω*, and the phase shift between the sinusoidal stress and the displacement as a function of oscillation frequency, of a particle are as follows:

$$A\left(\omega \right)=\frac{A_{L}k_{\mathrm{ot}}}{\sqrt{{\left(k_{\mathrm{ot}}+k\right)}^{2}+{\left(6\pi \eta \omega \right)}^{2}}}$$ (21)

$$\delta \left(\omega \right)=\mathrm{ta}n^{-1}\left(\frac{6\pi \eta \omega }{k_{\mathrm{ot}}+k}\right)$$ (22)

where *k* is the frequency dependent spring constant of the material. The shear elastic and shear viscus moduli are given by (Chiang et al., 2011):

$$G^{\prime }\left(\omega \right)=\frac{k_{\mathrm{ot}}}{6\mathrm{\pi r}}\frac{A_{L}}{A\left(\omega \right)}\cos\left(\delta \left(\omega \right)-1\right)$$ (23)

$$G^{\prime \prime }\left(\omega \right)=\frac{k_{\mathrm{ot}}}{6\mathrm{\pi r}}\frac{A_{L}}{A\left(\omega \right)}\sin\left(\delta \left(\omega \right)\right)$$ (24)

The interior of cells are often nonequilibrium environments due to intracellular activities. Passive and active microrheology have been combined to investigate non-thermally driven events in cells, such as motor protein activities, environmentally dependent stress, and cytoskeletal remodeling (Bursac et al., 2005; Mizuno et al., 2008; Wei et al., 2020; Hurst et al., 2021). A number of works have discussed nonlinear microrhology in detail (Squires and Brady, 2005; Squires, 2008; DePuit et al., 2011; Sriram and Furst, 2012; Hsiao et al., 2014).

OT have been widely used for microrheological measurements due to its versatility and wide frequency range. They can tweeze objects ranging from nanometers to micrometers with a force range of 0.1–100 pN (Furst, 2005). They can probe actively with an optical trap force or passively with a probe that displays thermal fluctuation. OT can also be configured to operate in oscillating mode or stretching mode. Frequencies on the order of 10^7^ Hz have achieved with OT (Taylor et al., 2013). Both active methods of OT and MT allow multiple-particle and single-particle measurements. However, one drawback of OT is the high intensity at the focus of the laser beam, which may overheat and damage the tissues or cells. MT does not typically have sample overheating problems and can be minimally invasive for biological samples. In addition, electromagnets can be thermally isolated, and the high-frequency oscillatory fields can be avoided in MT experiments to probe entirely non-invasively. MT operates with a broader force range from 10^−3^–10^2^ pN than OT, but is not as versatile as OT. MT has been used to actively probe the interior of cells (Robert et al., 2010; Lévy et al., 2011) and manipulate whole cells (Liu et al., 2009). Microrheological techniques, especially VPT, OT, and MT, have all been extensively used in characterizing biological samples due to their non-invasiveness, traceability, and their ability to relate local dynamics to spatial information. The rest of this article explores recent advancement of microrheological investigations in different mammalian organ systems, including associated cells, tissues, and biofluids.

### Operation Range of Microrheological Techniques

Conventional rheometry typically operates within the frequency range of 10^0^–10^2^ Hz. Common DLS can probe in a broader range of 10^−1^–10^3^ Hz, and Krajina et al. extended this frequency range to 10^−1^–10^6^ Hz (Krajina et al., 2017). However, DLS is limited to transparent and dilute samples. DWS requires probe particles for multiple scattering events and is more sensitive to particle motion, extending light scattering to measuring opaque samples at higher frequencies. DWS has been shown to have a broad frequency range of 10^0^–10^7^ Hz (Mason et al., 1997b). Both DLS and DWS are essentially bulk methods, providing an average sample measurement. Additionally, DWS and DWS usually require dedicated instruments, and the measurements typically rely on the setup. In comparison, VPT has been widely used for passive microrheological measurements for its relatively simple experimental setup, widely available tracking algorithms and software. The benefits, limitations, and operating regimes of these microrheological techniques are summarized in Table 1.

### Experimental Techniques in Biological Microrheology

**FIGURE 3:**
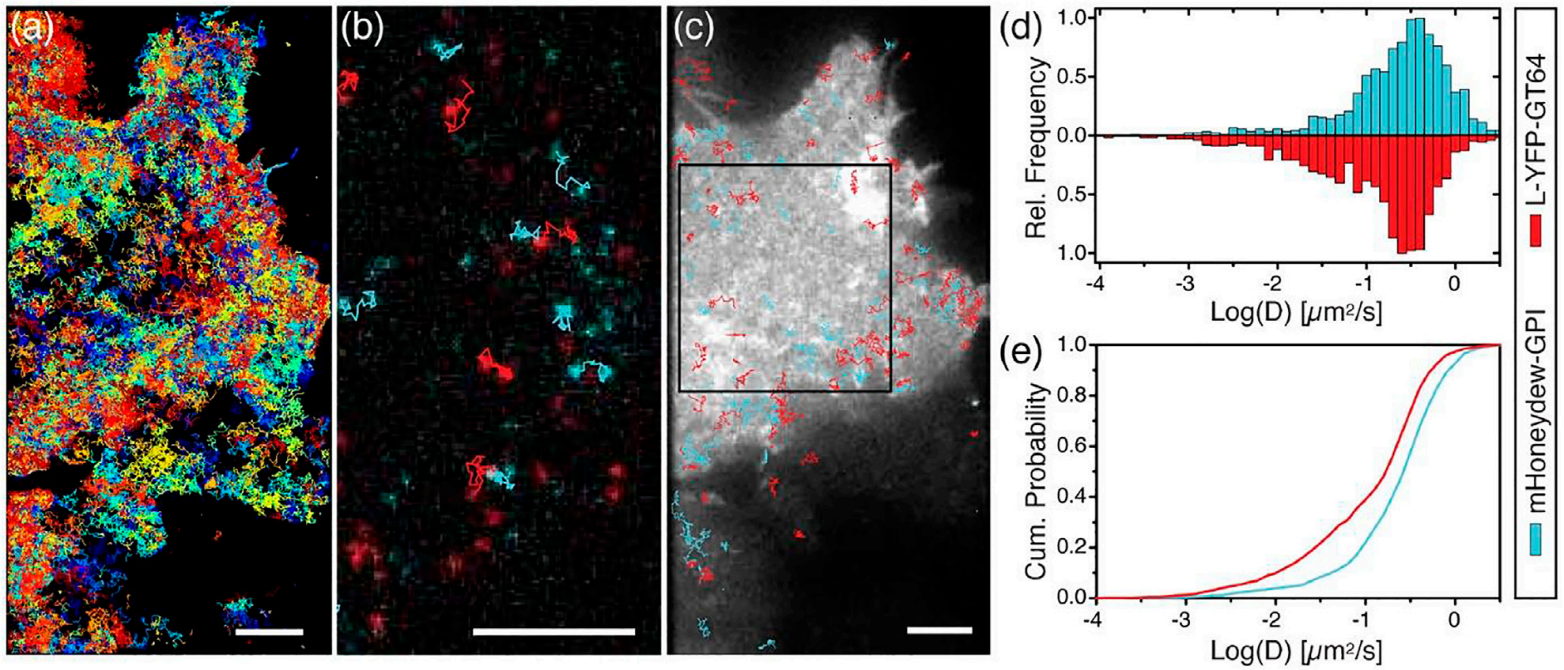
Single particle tracking of human bone osteosarcoma (U2OS) cells. **(A)** Fluorescent image of cells expressing L-YFP-GT46 (red-tracks) overlaid with tracks >5 frames. **(B)** Dual-color single particle tracking experiment. Image is a fluorescence micrograph showing tracks between two time points ∼0.75 s apart. **(C)** Fluorescent image of the YFP fluorescence of the cell overlain with blue and red dual fluorescent tracks with lengths of 30–40 frames. **(D)** Frequency histogram of log of diffusion coefficients for each probe type. **(E)** Cumulative probability plot for the diffusion coefficients. Scale bars are 5 microns. Figure adapted from Albrecht, David, et al. “Dual Color Single Particle Tracking *via* Nanobodies.” Methods and Applications in Fluorescence, vol. 3, no. 2, 2015, p. 024001., doi:10.1088/2050-6120/3/2/024001. Original work is available for use under the Creative Commons CC BY 3.0 License.

Synthetic particles, intracellular organelles and proteins have been used as probes in bio-microrheology. Synthetic probes are usually washed and sonicated prior to mixing with samples (Larsen and Furst, 2008; McGlynn et al., 2020). For intracellular measurements, cells are typically allowed to adhere to the bottom surface of a petri dish or well plate (Wirtz, 2009). OT can directly use the endogenous cellular components as probes. However, these natural probe size may vary and should be measured, typically through image analysis (Bertseva et al., 2012; Waigh, 2016). Intracellular granules, such as protein, can also serve as the probes for VPT (Figure 3) (Mak et al., 2014; Albrecht et al., 2015). Magnetically labeled endosomes have also been used as the probes for MT (Wilhelm et al., 2003b). Tipically, exogenous probes are introduced into cells through endocytosis, microinjection, and electroporation (Tseng et al., 2002; Weihs et al., 2006; Mandal et al., 2016; Katrukha et al., 2017). Considerations of particle toxicity and binding with proteins are discussed by Ehrenberg et al. (Ehrenberg and McGrath, 2005). As discussed in Section 2.1.4, the size of probes relative to their environment can affect their mobility. A scaling theory of particle mobility in different size regimes was initially developed by Rubinstein’s group (Cai et al., 2011) and discussed in the review by Waigh (Waigh, 2016). This theory provides a reference for probe size selection. Length scale dependent microrheology has been performed by multiple groups (Lu and Solomon, 2002; Lai et al., 2009a; Weigand et al., 2017). As biological samples can be spatially heterogeneous, TPM that are insensitive to heterogeneity can be used in these environments. Intracellular MPT and TPM experiment method and principles is discussed in these reports (Lau et al., 2003; Hoffman et al., 2006; Crocker and Hoffman, 2007). For comprehensive experiment method and principles in cells and biomaterials, including sample preparation and data interpretation, we direct the readers to these excellent reviews (Kasza et al., 2007; Wirtz, 2009; Waigh, 2016; Joyner et al., 2020; McGlynn et al., 2020).

## Microrheological Approaches to Evaluate Mechanical Properties of Cells, Tissues, and Biofluids

### Digestive System

The human digestive system includes organs and chemically corrosive fluids that break down and maneuver nutrients throughout the body, then to tissue for metastasis. The digestive system consists of the gastrointestinal tract and accessory digestive organs. The gastrointestinal tract is the main pathway in which food is broken down, nutrients are extracted, and waste is excreted. The mucus secreted by ciliated cells aids in digestion and forms a barrier to protect internal organs from the harsh pH of the esophageal, stomach, and intestinal environments (Johansson et al., 2013). Some accessory organs of the digestive system provide the system with mechanical digestion, other accessory organs secrete fluids and bile that aid in chemical digestion, such as the saliva glands, liver, pancreas, and gallbladder. Measuring the mechanical properties of these tissues and fluids helps us understand the pathology and mechanisms of digestive diseases, cancers, and disorders, which further facilitates the development of therapeutics.

Pancreatic cancer is of particular interest due to its high fatality rate, poor prognostic ability, and low early detection rate (Goral, 2015; Ansari et al., 2016). Pancreatic disease and tumor growth are influenced by various extracellular factors, such as bidirectional stromal communication in pancreatic tissue and the resulting extracellular matrix (ECM) remodeling. Specifically, cancer cells secrete proteolytic enzymes to degrade the surrounding ECM to gain mobility to grow and spread. In turn, increasing fibroblast proliferation induces an increase in ECM stiffness to contain tumor growth and metastasis. This stromal containment is particularly prominent in pancreatic ductal adenocarcinoma. VPT has been used to show ECM dynamics upon the invasion of human pancreatic epithelial carcinoma cells (cell line PANC-1) (Jones et al., 2014; Larin et al., 2015). VPT has also been used to measure the mechanical properties of the ECM of a 3D tumor model cultured with PANC-1 cells (Jones et al., 2014). Results showed that G’ (1 rad/s) of regions with no cancer cell invasion remains in the order of 10 Pa for 4 days. However, cancer cell invaded regions showed significant softening and liquefication by day four. Understanding both directions of cell-environment interactions is essential in understanding the progression of pancreatic tumors. In a recent investigation into these mechanical relationships using VPT method, PANC-1 cells and MRC-5 (normal human fibroblast) cells were cultured in collagen I ECM to build pancreatic models: PANC-1 homoculture, PANC-1/MRC-5 co-culture, and cell-free control (Jones et al., 2017). The resulting MSDs of the coculture and the control were similar, but the MSD of the PANC-1 homoculture revealed a high degree of structural heterogeneity. G′ of the control was approximately 0.7 Pa (reported at 1 rad/s), G′ of the PANC-1 homoculture was significantly lower (∼0.1 Pa), indicating degradation of the ECM caused by the invasion of cancer cells; however the significant decrease in G’ was hindered by the presence of fibroblasts (∼0.5 Pa) (*see* Table 2).

**TABLE 2:** Microrheological properties of mammalian cells and tissue of the digestive system measured by various microrheological techniques.

Cell/tissue	Storage modulus (G′)	Loss modulus (G″)	Complex modulus (|G*|)	Scaling of storage modulus	Scaling of loss modulus	Scaling of complex modulus	Creep compliance (*J*)	Viscosity (η)	Pore size (ξ)	Reference
PANC-1	∼0.90–1.3 Pa (1 rad/s) VPT									Cramer et al., 2016 Cramer et al. (2017)
Chemo-resistant subline PANC1OR	∼0.25–1.1 Pa (1 rad/s) VPT
COL1 ECM upon PANC-1 invasion	∼0.10 Pa (10 Hz) VPT									Jones et al. (2017)
COL1 ECM with embedded PANC-1 and MRC-5	∼0.50 Pa (10 Hz) VPT								1.72 μm VPT
COL1 ECM upon PANC-1 spheroid invasion after 4 days	∼0.0005 Pa (1 rad/s) VPT									Jones et al. (2014)
Human Saliva	∼0.02 Pa OT	∼0.007 Pa OT						1.3–3.0 × 10^−3^ Pa s OT		Teubl et al. (2018)
Mouse gastrointestinal mucus (wild type)									148 nm (mean) VPT	Difato et al. (2011)
Mouse gastrointestinal mucus (rCYSx12-enriched mucus from transgenic mouse)									118 nm (mean) VPT	
Purified porcine MUC5AC pH7 (30–50 mg/ml)			∼2–5 Pa VPT					∼0.001–0.6 Pa s VPT		Georgiades et al. (2014)
Purified porcine MUC2 pH7 (50–100 mg/ml)			∼0.67–5 Pa VPT					∼0.002–5.0 Pa s VPT
Purified porcine MUC5AC pH1 (10 mg/ml)			∼3.0 Pa VPT					∼3.0 Pa s VPT
Purified porcine MUC2 pH1 (10 mg/ml)			∼0.67 Pa VPT					∼0.82 Pa s VPT

Microrheological results can uncover the evolution of the tumor microenvironment and aid in the development of pancreatic cancer treatment. In a recent work by Cramer et al. (Cramer et al., 2017), rheological techniques were employed to show that photodynamic therapy targets invasive and chemoresistant pancreatic cells. Similar 3D co-cultures containing PANC-1, MRC-5, and BxPC-3 (human pancreatic epithelial carcinoma) cells were grown in type I collagen (COL1) and Matrigel, respectively. Bulk rheological results showed both hydrogels were soft gels with the storage modulus dominating over the loss modulus. However, Matrigel was significantly stiffer with a G’ ∼ 90 Pa, than COL1 at 1 mg/ml with a G’ ∼ 5 Pa (both reported at 1 Hz). VPT results of both hydrogels showed higher G′ in the upper focal planes and lower G′ in the lower focal planes. In addition, the drug-resistant subline of PANC-1, PANC1OR showed lower G’, indicating more severe ECM degradation than the other PANC-1 cell culture.

Saliva and digestive mucus are complex biological fluids that act as protective barriers to organs, the first line of defense against pathogens, and lubrication for the food digestion process. The primary solid components of mucus include glycoproteins, mineral salts, lipids, and DNA. The glycoprotein mucin is the main component of mucus that forms an entangled network through covalent and non-covalent bonds, accounting for about 5% of the total weight of normal mucus (Allen et al., 1993). Microrheology has often been utilized to investigate these fluids. Recently, investigations of the relation between nanoparticles’ physicochemical properties and their mobility in saliva have been carried out *via* DLS and OT (Teubl et al., 2018). Using DLS, the diffusivities of aminated, carboxylated, and non-functionalized polystyrene particles were determined to evaluate their mobility. Results showed that non-functionalized nanoparticles with a negative charge remain stable in saliva. Furthermore, the bulk rheological measurements of shear moduli were significantly higher than microrheological measurements. This discrepancy suggests the interstitial spaces of the mucus network are filled with a fluid with a viscosity close to water. Understanding saliva and particle dynamics opens the door for payload delivery treatment for oral inflammatory diseases.

Recent work with particle tracking microrheology has shown that enrichment with certain types of mucin structural domains causes mucus to be stiffer and less permeable, potentially becoming a more robust barrier for invading pathogens (Demouveaux et al., 2019). Microrheology has been employed in the development of treatment for mucus-based infection through intentional stiffening of the mucus-gel network to prevent penetration of undesired microbes and debris (Qin et al., 2008; Demouveaux et al., 2019). A VPT investigation of duodenum mucin (MUC5AC) and gastric mucin (MUC2) showed these mucins at pH = 7 formed self-assembled networks above the semi-dilute overlap concentration (c∗) with scaling of viscosity η∼c^1/2^ in agreement with the Fuoss law for semi-dilute linear flexible polyelectrolytes. While above the entanglement concentration (c_e_), a noticeable increase in the viscosity scaling was observed, with η∼c^3.92±0.38^ for MUC5AC and η∼c^5.1±0.8^ for MUC2 (Table 2). At pH = 1, both mucin solutions gelled permanently due to the creation of cross-links between neighboring self-assembled chains. These results revealed the underlying mechanisms in which glycoproteins polymerize and form gels that protect the epithelial cells of the high-pH gastrointestinal system (Georgiades et al., 2014).

### Respiratory System

The main functions of the respiratory system are inhalation of oxygen and gas exchange that occurs *via* diffusion in small, sac-like structures called alveoli in the lungs. The main organs of the respiratory system are mostly made of secretory epithelial tissue, providing a layer of mucus along the respiratory system that serves to filter dust out of inhaled gas to protect the tissue from infection and lubricate the airway (Zanin et al., 2016). Measuring the mechanical properties of alveolar and bronchial lung cells is essential in understanding the structural integrity of the lung during cellular maintenance activities, cyclical mechanical activity (e.g., breathing), and lung disease progression. Both active and passive microrheology have been utilized to investigate the relationship between respiratory pathologies and tissue/mucus viscoelastic properties.

The change in the intracellular and extracellular mechanical properties from healthy to cancerous tissue has been investigated *via* VPT (Panzetta et al., 2017). Comparing the MSDs of probe particles in the cytoplasm between healthy cells and cancerous cells suggested cell softening is associated with cancerous transformation. In contrast, the MSDs in healthy and cancerous ECM showed tissue stiffening during malignant progression, resulting from increased integrin activity and focal adhesion signaling to promote the proliferation of cancerous cells. A 2020 investigation of *Pseudomonas aeruginosa* (PA) infection’s effect on health was conducted on a prolonged acute murine PA-infection model (Radiom et al., 2021). The microrheological properties of the lung homogenates from infected mice were evaluated using VPT. Results showed that the three lung homogenates out of the 12 of the infected samples displayed plateau moduli (*G*_*0*_) within the range of 2.48–3.03 Pa, with a diffusive exponent (*α*) below 0.2, and a mesh size (*ξ*) below 130 nm. The viscosity (*η*) of the other nine samples was determined to be 2.41–6.22 mPa·s. The diffusive exponents (*α*) were observed to decrease with increasing values of colony forming unit (CFU), *Pseudomonas* quinolone signal (PQS), and 2-heptyl-4-quinolone (HHQ) that participates in PA transcription.

**FIGURE 4:**
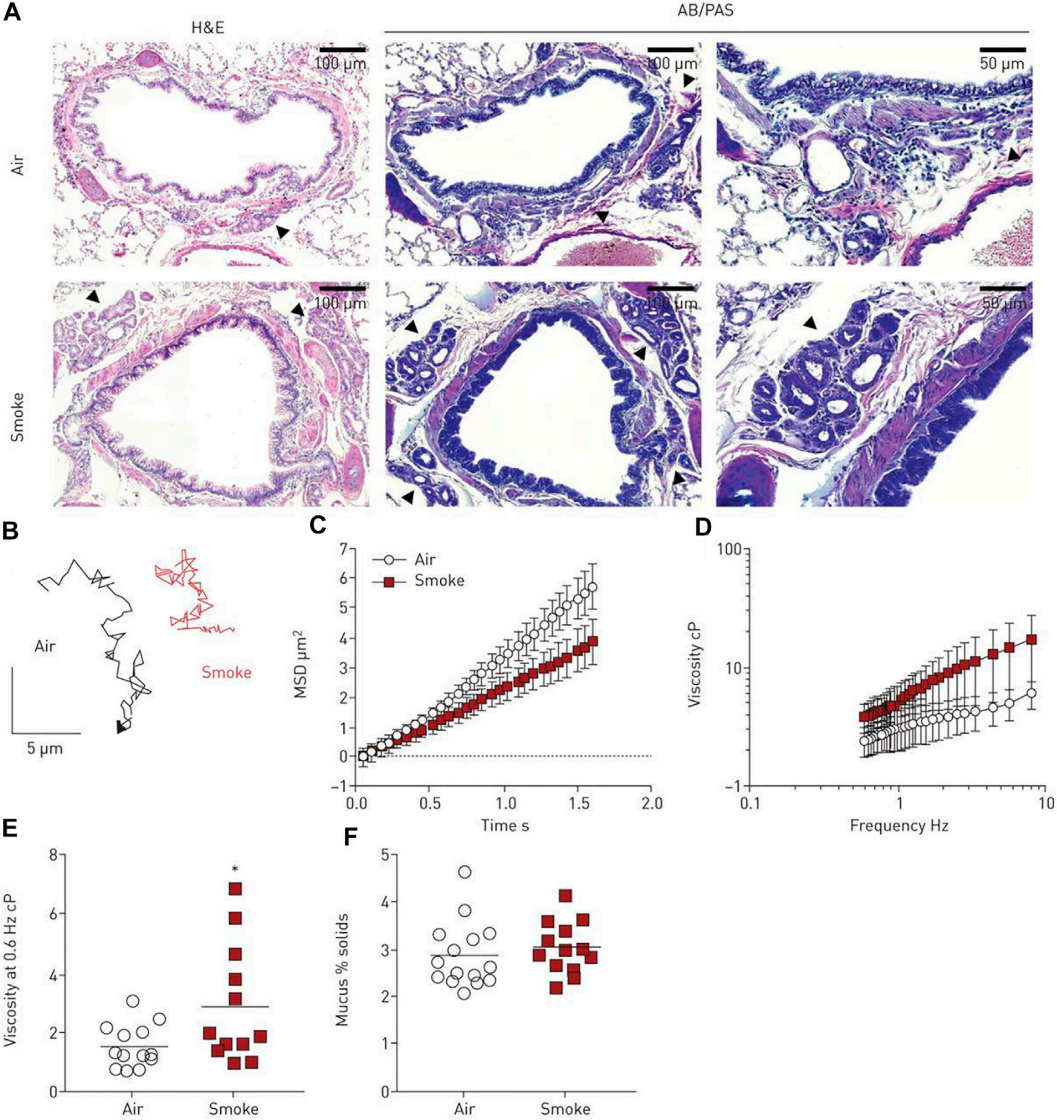
Mucus viscosity increases with smoke exposure in ferrets. **(A)** Histology of tracheal and lung tissue samples from control and smoke-exposed ferrets. Submucosal glands are indicated by the black arrows. **(B)** Sample Brownian motion VPT tracks of 500 nm diameter tracer probes imbedded in the mucus of both ferret groups. **(C)** MSD as a function of lag time and **(D)** effective viscosity as a function of frequency for the control and smoke-exposed ferret groups. **(E)** Effective viscosity of both mucus groups at a frequency of 0.6 Hz. **(F)** Percent solid content of mucus groups, calculated from wet and dry weights. *N* = 12–15 ferrets per group, *p* < 0.05 (unpaired Student’s t-test). Figure reproduced with permission of the ^©^ ERS 2022: European Respiratory Journal Jan 2020, 55 (1) 1900419; DOI: 10.1183/13993003.00419-2019.

Additionally, rheological properties of lung and airway mucus have been tied to disease states for many respiratory ailments: cystic fibrosis, laryngeal tuberculosis, bronchitis, asthma, chronic obstructive pulmonary disorder (COPD), voice disorders, and lung plaque buildup from smoking (Lai et al., 2009b; Lin et al., 2020; Peters et al., 2021). The microrheology of pulmonary mucus also directly affects the movement of this protective barrier throughout the lung and airway and, therefore, its effectiveness as a protective barrier. Respiratory mucus originating from the vocal cords plays an essential role in voice disorder pathology in smokers. Recently, researchers investigated laryngeal mucus collected from the vocal folds of smokers and non-smokers using both VPT and bulk rheology (Peters et al., 2021). Both methods uncovered gel-like characteristics in all groups; however, mucus rigidity varied depending on the smoking habits of the subject. Mucus samples were categorized into three groups, where Group *a* is solid-like within the range of measurement, Group *b* shows G″ dominance at *ω* > 10 s^−1^, and Group *c* shows G′ dominance at *ω* > 10 s^−1^. The average shear moduli decreased from Group *a* (∼10^0^–10^1^ Pa at 1 Hz) over Group *b* (∼10^−1^–10^0^ Pa at 1 Hz) to Group c (∼10^−2^–10^−1^ Pa at 1 Hz). The shear moduli measured by bulk rheology were one magnitude higher than microrheological results, but both techniques showed G’ > G″ at rest. It was found that low shear moduli in Group *c* were associated with smoking as a result of a loose mucin network and varying hydration levels. Researchers suggested that this work could be used as a basis for the engineering of artificial vocal-chord mucus to relieve patients of irritating symptoms due to smoking. Additionally, the mucociliary transport of mucus throughout the airway can also be examined using VPT (Lin et al., 2020), uncovering the mechanisms of throat-clearing problems in the larynx of smoking individuals. VPT measurements revealed an elevation in the viscosities of mucus from smoke-exposed ferrets and mucus derived from primary human bronchial epithelial cells from donors with chronic obstructive pulmonary disease (Figure 4). Passive microrheology has also helped uncover the morphogenesis in the prenatal development of lung. In a recent investigation, contractions involved in airway peristalsis were found to be related to the development and fluid transport within the embryonic lung of prenatal mice (Bokka et al., 2015). Microrheology is particularly useful in this interrogation; by injecting the probe particles into the intraluminal space of the terminal buds, the viscosity of embryonic lung lumen fluid was measured for the first time, which is surprisingly a Newtonian fluid with a viscosity of 0.016 ± 0.008 Pa·s. The analysis of particle diffusion showed that airway peristalsis in the embryonic airway induces a dramatic increase in the fluid transport efficiency, suggesting its involvement in stimulating and regulating morphogenesis is not only through tissue stretching, but also morphogen transport.

Additionally, the effect of size and surface functionalization of respiratory infection-treating drug-delivering nanoparticles on their ability to penetrate healthy mucus have been investigated using VPT. Employing both VPT and bulk rheology, researchers determined that densely polyethylene PEGylated nanoparticles (100 nm) can rapidly penetrate airway mucus due to reduced mucus adhesion (Schuster et al., 2013). A 2012 investigation used OT and capillary penetration tests to evaluate the penetration of nanoparticles coated with PEG and chitosan through pulmonary mucus and hydroxyethylcellulose (HEC) hydrogel models. Combined with Cryo Scanning Electron Microscopy (Cryo-SEM), results showed the highly heterogeneous structure of the pulmonary mucus substantially obstructs particle penetration (Kirch et al., 2012). VPT has also been employed to characterize artificial sputum medium (ASM) that recreate a microenvironment resembling sputum from patients with cystic fibrosis (CF) (Tan et al., 2020). The shear moduli of ASM at varying concentrations determined by VPT with 1 µm probe particles showed agreement with microrheological measurements. Still, all microrheological measurements are lower than bulk rheological measurements within one order of magnitude. The transition of the MSD from short to long lag times is the characteristic length describing the cage size in which particles are trapped inside. The characteristic lengths of the mucus models were identified to aid in the development of treatment for CF that can penetrate through the thick mucus layer. A 2021 report also employed VPT to aid in the design of synthetic mucus with microrheological properties comparable to native healthy and asthma mucus (Song et al., 2021). As infection by influenza A virus was introduced to the asthma-like synthetic mucus rich in MUC5AC, the increase in the diffusion rate in the mucus medium indicated impairment of its barrier function. This investigation presented a biomaterial model that can be precisely controlled to mimic native mucus, whether healthy or diseased. These controllable models can be used to understand airway dysfunctions like asthma.

When combining AFM, VPT, OT, and cryo-SEM, researchers uncovered the heterogeneity of pulmonary mucus. Of these techniques OT was determined to provide the broadest range of mucus’ mechanical and structural properties, including pore size, viscosity, rigidity, and particle mobility. Due to limitations in collecting lower airway and lung mucus, *in vitro* cell culture has been used to study the mechanical properties and subsequent drug delivery treatment of these respiratory biofluids. To validate human bronchial epithelial (HBE) models, Jory et al. collected HBE cells during fiber optic bronchoscopy and cultured them for mucus secretion. OT was used to measure the microrheological properties of this mucus to compare with *ex vivo* mucus. They found that *ex vivo* mucus display more viscous behavior and shear moduli several orders of magnitude lower than the cultured mucus on the epithelium. Additionally, the microrheological properties of *ex vivo* mucus showed high variance between patients, which was caused by the varying cell debris collected along with the sample. The cultured mucus showed higher consistency between batches; however, its mechanical properties decreased with the distance from the epithelium (Jory et al., 2019). The microrheological measurements of the respiratory systems are listed in Table 3.

**TABLE 3:** Microrheological properties of mammalian cells and tissue of the respiratory system measured by various microrheological techniques.

Cell/tissue	Storage modulus (G′)	Loss modulus (G″)	Complex modulus (|G*|)	Scaling of storage modulus	Scaling of loss modulus	Scaling of complex modulus	Creep compliance (*J*)	Viscosity (η)	Pore size (ξ)	Reference
Healthy human sputum	∼0.04 Pa (10^2^ Hz) DLS	∼0.18 Pa (10^2^ Hz) DLS		${\text{G}}^{\prime }∼\;\omega$ DLS						Cai et al. (2021)	
Human cystic fibrosis sputum	∼0.5 Pa (1 Hz) DLS	∼0.5 Pa (1 Hz) DLS		${\text{G}}^{\prime }∼\;\omega^{\frac{1}{4}},\;\omega^{\frac{1}{2}},\;\omega^{\frac{2}{3}}$ DLS									
Normal human sputum								∼4 cP (0.6 Hz) VPT		Lin et al. (2020)	
Sputum of healthy smoker								∼15 cP (0.6 Hz) VPT					
Chronic obstructive pulmonary disease sputum								∼800 cP (0.6 Hz) VPT					
Human laryngeal mucus (Group a)	12.28 ± 12.89 (0.6 Hz) VPT	4.19 ± 4.60 (0.6 Hz) VPT								Peters et al. (2021)	
Human laryngeal mucus (Group b)	0.80 ± 0.43 (0.6 Hz) VPT	0.28 ± 0.15 (0.6 Hz) VPT											
Human laryngeal mucus (Group c)	0.05 ± 0.02 (0.6 Hz) VPT	0.03 ± 0.00 (0.6 Hz) VPT											
Human cystic fibrosis sputum	4.9 ± 2.2 Pa (1 rad/s) MRS^a^	3.6 ± 2.4 Pa (1 rad/s) MRS^a^						67 ± 34 Pa s (1 Hz) MRS^a^		Radiom et al. (2021)	
Lung homogenates of 12 independent mice								2.41 ± 0.75 to 6.22 ± 6.03 mPa s VPT	123 ± 27 to 208 ± 72 nm VPT	Murgia et al. (2020)	
Mucus collected from human endotracheal tubes									400–500 nm VPT	Song et al. (2021)	
*In vitro* BCi-NS1.1 mucus									∼300–400 nm VPT				
MUC5B hydrogels									∼200–400 nm VPT				
MUC5AC gels									∼100 nm VPT				
MUC5B/MUC5AC (75:25)									∼100 nm VPT			
Embryonic mouse lung lumen								0.016 ± 0.008 Pa s		Bokka et al. (2015)		
*Ex vivo* human bronchial epithelial mucus	∼0.01 Pa (1 Hz) OT			G’ ∼ ω^0.85^ OT						Jory et al. (2019)	
Mucus of human bronchial epithelium culture (10 μm from epithelium)	∼2.3 Pa (1 Hz) OT			G’ ∼ ω^0.27^ OT									
Mucus of human bronchial epithelium culture (30 μm from epithelium)	∼0.3 Pa (1 Hz) OT			G’ ∼ ω^0.78^ OT									
Interstitial fluid of human respiratory mucus								∼0.003 Pa s (1 rad/s) VPT		Schuster et al. (2013)	
Human respiratory mucus (100 nm PS-PEG probe)								∼0.05 Pa s (1 rad/s) VPT					
Human respiratory mucus (200 nm PS-PEG probe)								∼0.12 Pa s (1 rad/s) VPT					
Human respiratory mucus (500 nm PS-PEG probe)								∼3.5 Pa s (1 rad/s) VPT					

a Magnetic rotational spectroscopy.

### Nervous System

The human nervous system is a highly-complex pathway of neurons that receives signals from external stimuli, processes information, and outputs commands to different tissue of the human body. The nervous system consists of the central nervous system (CNS) and the peripheral nervous system (PNS). The PNS consists of the somatic nervous system (SNS) and the autonomic nervous system (ANS). The CNS, including the brain and the spinal cord, acts as the body’s main control center and processing domain. The brain is of particular interest in microrheology, as its operation is still poorly understood due to technical and philosophical barriers (Pagán, 2019). The brain is also the host to many under-studied yet detrimental disorders, including Alzheimer’s disease, Parkinson’s disease, brain cancer, and various mental illnesses/mood disorders.

The nervous system is challenging to explore due to its inherent complexity and heterogeneity, the ethical considerations of investigating the human brain, and the physical challenges of taking rheological measurements in the neuronal systems. Microrheology shows the potential for filling the knowledge gap in the microscopic mechanical structure of the brain. This understanding can further facilitate the improvement of neural technologies. For example, successful brain-like biomaterials can be used for brain-computer interfaces and neural tissue engineering (Axpe et al., 2020). Additional innovations unlocking the heterogeneity of the brain may accomplish drug delivery across the blood-brain barrier (Liebner et al., 2018), brain cancer treatments (Chen et al., 2018; Alibert et al., 2021), and development in cranium protective equipment for traumatic brain injury prevention (Mazumder et al., 2013). In a recent report, VPT was combined with machine learning to push the boundaries of passive microrheology. By analyzing large datasets of nanoparticle trajectories, the viscosity of artificial cerebrospinal gels was predicted with a 0.75 recall score; and the *in situ* particle size was predicted with a 0.90 recall score. The model was also validated with the effect of protein adherence and reduction of protein adherence by PEG-surface modification (Curtis et al., 2019).

Active microrheology has also been used to interrogate the highly complex, heterogeneous cytoskeleton network within neurons. MT was used to analyze the mechanical properties of crosslinked microtubule networks formed by bovine and porcine brain-derived tubulins. The microtubule networks were formed with biotinylated tubulin relative to total tubulin at molar percentages of 12.5, 25, and 50%. Results revealed that all microtubule networks were highly heterogeneous and force-dependent, displaying reversible elasticity, meaning that they become stiffer in response to small forces and softer in response to larger forces at shorter time scales (Yang et al., 2013). On a longer time scale of 5–10 s, the creeping flow behavior of the microtubule networks was observed and attributed to magnetic force-induced bond unbinding. The authors suggest that this model system serves as a test platform for future research to understand intracellular cytoskeleton remodeling in response to external loading, an important concept in biophysics and tissue structure/behavior.

Interrogating the mechanical properties of brain tumor cells allows us to understand the cellular mechanics of brain cancer progression and metastasis. The change in the mechanical properties of cancerous cells and tissue can also serve as a diagnostic and prognosis tool. A recent investigation of glioma cells employed a single-cell parallel plates rheometer for whole-cell measurement and OT for intracellular measurement (Alibert et al., 2021). In the whole-cell measurement, human glioma cell line U373, a type of grade III astrocytoma, was found to exhibit a |G*| of ∼200 Pa (*ω* = 1 Hz); cell line U87, a type of grade IV glioblastoma, was found to have a G* of only half of the former. In contrast to whole-cell rheology, intracellular measurements by OT showed |G*| of U373 cells within the range of 20–30 Pa and |G*| of U87 within the range of 30–40 Pa (Table 4). These findings were attributed to cortex softening and intracellular stiffening of U87 grade IV glioblastoma cells. Like many other types of cancer, glioma brain tumors respond directly to extracellular mechanical queues and structure their microenvironments accordingly. Therefore, it was shown that investigation of the mechanical properties of glioblastoma cells may be developed as a diagnostic method.

**TABLE 4:** Microrheological properties of mammalian cells and tissue of the nervous system measured by various microrheological techniques.

Cell/tissue	Storage modulus (G′)	Loss modulus (G″)	Complex modulus (|G*|)	Scaling of storage modulus	Scaling of loss modulus	Scaling of complex modulus	Creep compliance (*J*)	Viscosity (η)	Pore size (ξ)	Reference
Primary astrocytes from E17 rat embryos	∼43 Pa (1 Hz) OT	∼14 Pa (1 Hz) OT								Alibert et al. (2021)
Rat glioblastoma cell line F98	∼22 Pa (1 Hz) OT	∼5 Pa (1 Hz) OT								
Human glioma cell line U373 (grade III astrocytoma)	∼20 Pa (1 Hz) OT	∼18 Pa (1 Hz) OT	∼28 Pa (1 Hz) OT							
	∼210 Pa (1 Hz) Single-cell microplate	∼180 Pa (1 Hz) Single-cell microplate	∼80 Pa (1 Hz) Single-cell microplate							
Human glioma cell line U87 (grade IV glioblastoma)	∼36 Pa (1 Hz) OT	∼30 Pa (1 Hz) OT	∼18 Pa (1 Hz) OT							
	∼100 Pa (1 Hz) Single-cell microplate	∼80 Pa (1 Hz) Single-cell microplate	∼40 Pa (1 Hz) Single-cell microplate							
Rabbit vitreous	0.014 ± 0.026 Pa (10 rad/s) MT	0.006 ± 0.009 Pa (10 rad/s) MT								Pokki et al. (2015)
	0.14 ± 0.26 Pa (300 rad/s) MT	0.11 ± 0.23 Pa (300 rad/s) MT								
*Ex vivo* human vitreous							∼750 m/N (1 s) OT			Watts et al. (2014)
*Ex vivo* porcine vitreous							∼100 m/N (1 s) OT			
*Ex vivo* bovine vitreous (100, 200, and 510 nm PS-PEG probes)	∼0.04 Pa (2 rad/s)							∼0.110 Pa s VPT		Xu et al. (2013)
*Ex vivo b*ovine vitreous (1000 nm PS-PEG probes)	∼2.8 Pa (2 rad/s)							∼1.1 Pa s VPT		

**FIGURE 5:**
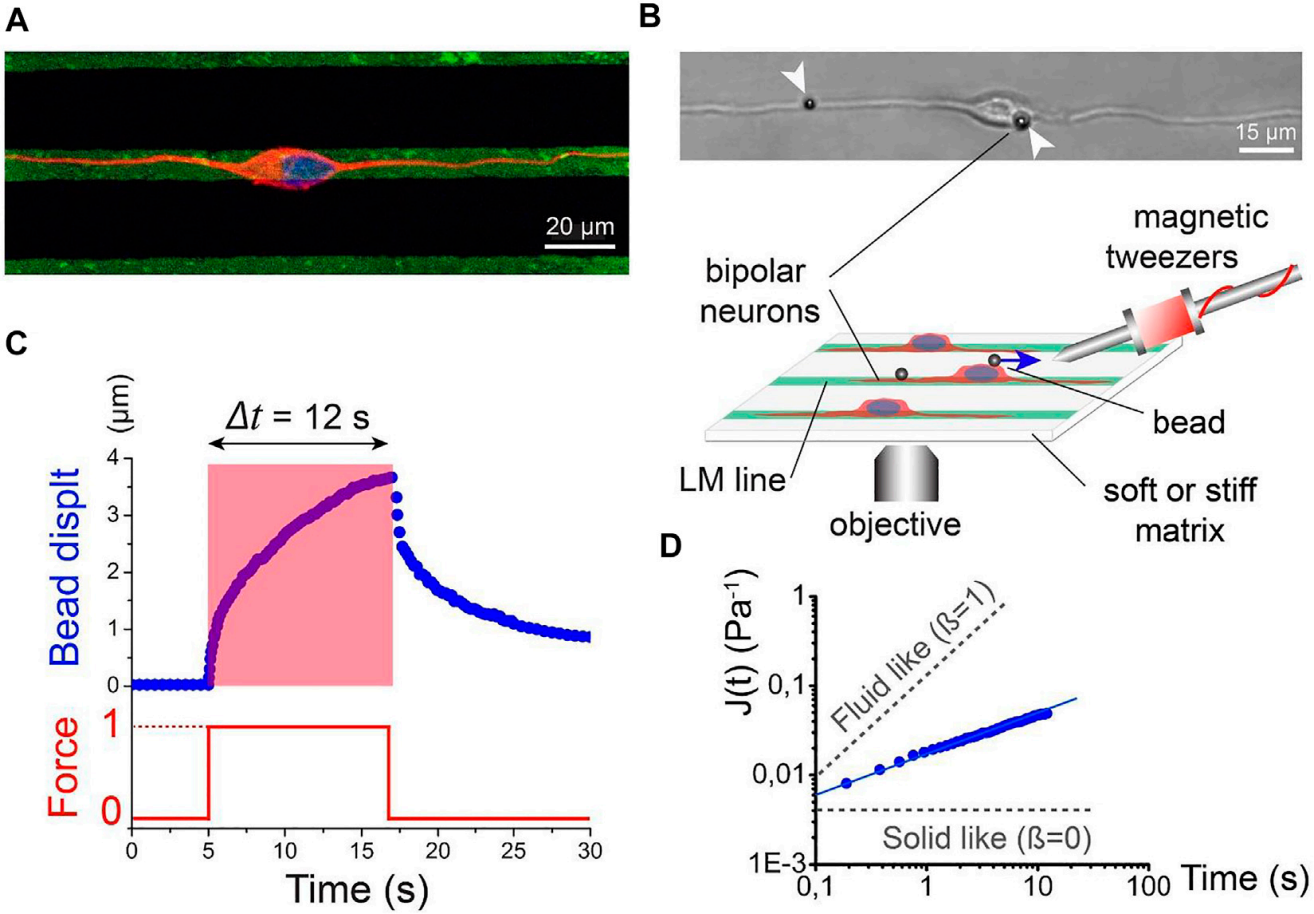
Active rheology of cortical neuronal microcompartments. **(A)** Immunofluorescence micrograph of nucleus (blue) and microtubules (red) of a bipolar neuron attached to laminin lines (green). Scale bar: 20 microns. **(B)** Schematic of magnetic tweezer rheological set up. Bipolar morphology of cortical neurons was achieved by growth on laminar strips. A bipolar neuron with magnetic beads attached to the membrane is depicted by the DIC image. Scale bar: 15 microns. **(C)** Bead displacement as a function of time (blue line) in response to a step force input (red line). **(D)** Logarithmic of creep compliance *J(t)* as a function of time. The slope of the graph (beta) depicts solid-like and liquid-like behavior. Figure from Grevesse, T., et al. Opposite rheological properties of neuronal microcompartments predict axonal vulnerability in brain injury. *Sci Rep* **5,** 9475 (2015). https://doi.org/10.1038/srep09475 Original work is available for use under the Creative Commons CC BY 4.0 license.

In addition to cancer and neurodegenerative disease, microrheology can also be a valuable tool to investigate cellular responses to traumatic brain injuries (Grevesse et al., 2015). In this report, the mechanical responses of neuronal microcompartments were measured by subjecting them to external forces generated by MT. It was observed that the axons of cortical neurons from rat embryos were more viscous than the rest of the cell body as a result of a cytoskeleton adaptation to substrate stiffness. Additionally, the disproportionate energy dissipation of neurites in response to the external force was attributed to intracellular fluidization caused by cytoskeletal remodeling. The findings provide insight into the mechanisms of neuron pathology upon traumatic brain injury and facilitate the development of targeted axonal-repair treatments (Figure 5). Laser dissection has recently been combined with OT systems to explore the corrective regrowth of neural networks after ablation (Difato et al., 2011). Neurons of E18 rat hippocampi were allowed to form a connection with the ECM; then a laser dissector was used to damage a neurite while the tensile force change on the cytoskeleton was measured simultaneously, providing insights on the mechanics of neuronal damage and repair, as well as the functional contributions of neurons.

Fluids in accessory nervous system organs responsible for sensing can also be investigated using numerous microrheological methods. The potential for nanotechnology to aid in surgery, drug delivery, and diagnosis platforms in the human eye is a topic that has been increasingly explored. VPT has been used to interrogate the permeability of bovine vitreous. Two reports showed that particle mobility was suppressed by the selective permeability nature of vitreous humor, which was largely dependent on the electrostatic interactions between the biopolymer network and diffusing particles (Xu et al., 2013; Käsdorf et al., 2015). The movement of particles with a positively charged surface tended to be hindered, while negatively charged particles became diffusive when they were small enough (≤200 nm) and less concentrated. However, densely PEGylated particles exhibited rapid penetration through the network (Xu et al., 2013). In addition to passive techniques, active manipulating particles in the vitreous humor provides more insights for targeted drug delivery. OT has been used to assess the microrheological properties of rabbit vitreous humor. As a result, heterogeneous behavior was observed, with a mean storage modulus of 0.014 ± 0.026 Pa and a mean loss modulus of 0.006 ± 0.009 Pa (reported at 10 rad/s) (Watts et al., 2014). A 2015 investigation reported the compliance of artificial vitreous, *ex vivo* human vitreous, *ex vivo* porcine vitreous, and *in vivo* rabbit eyes measured by MT and AFM. The measured compliance of *ex vivo* human vitreous was about one order of magnitude higher than *ex vivo* porcine vitreous and artificial vitreous (Pokki et al., 2015). Another investigation of porcine vitreous *via* MT reported a linear velocity of 0.48 ± 0.12 μm/s for 500 nm particles under a gradient of ∼80 T/m, indicating an apparent viscosity of 0.8 Pa·s. However, the linear velocity of 1 and 2.7 µm particles drastically decreased to 0.11 ± 0.06 μm/s and 0.06 ± 0.06 μm/s, respectively (Table 4) (Qiu et al., 2014). These results suggest a ∼500 nm mesh size of the vitreous humor.

Further investigation of active delivery within the vitreous humor has been conducted by Wu et al., where micropropellers consisting of magnetic particles with a helical tail were driven by a rotating magnetic field to translate in the porcine vitreous humor (Wu et al., 2018a). The diffusion coefficients of perfluorocarbon-coated and uncoated micropropellers of various sizes were reported. The smallest micropropellers with a diameter of 300 nm were the most mobile with a diffusion coefficient ∼0.6 μm^2^/s, while 1 µm micropropellers were almost immobile. They also reported a speed of ∼10 μm/s for the perfluorocarbon coated micropropellers with a 500 nm diameter in the porcine vitreous humor.

Microrheological exploration in the brain reveals the complex microstructure and mechanical properties of healthy and malignant tissue/cells. In addition, OT-based force spectroscopy was used to monitor neuron activities in response to damage and identify neurites’ functional contributions. These microrheological advancements in the brain provide insight into brain disease progression, propelling the development of diagnostic/prognostic tools and effective treatment in the brain. Understanding the microstructure of the brain and functions of neurons also helps to develop artificial brain models that mimic brain environments that can serve as a testing platform. In the accessory nervous system organs like the vitreous humor of the eye, microrheology has been utilized to understand the pathology of ocular diseases such as cataracts, glaucoma, and vitreous humor degeneration. Microrheological quantification of the vitreous also helps to improve control and precision of ocular instrument, and can serve as a prognostic tool. Understanding the microrheological properties of vitreous humor’s heterogeneous environment is essential to optimize nanorobot size and speed for efficient propulsion (Wang et al., 2008).

### Circulatory System

The human circulatory system contains two main subsections: the cardiovascular system (a closed organ system) and the lymphatic system (an open system). The cardiovascular system is comprised of the heart, blood, and vessels; this system circulates nutrients and oxygen through the body, fights off disease, maintains the body’s homeostasis and pH levels, fights off diseases, and regulates inflammation (Silvestre-Roig et al., 2020). The heart, made of striated cardiac muscles, pumps blood around the body and is controlled by the autonomic nervous system. Blood, the human body’s only fluid connective tissue, consists of erythrocytes, plasma, white blood cells, and platelets. The main functions of blood are to carry oxygen from the lungs to the tissue, carry carbon dioxide from the tissue to the lungs to be expelled, and provide immune defense against pathogens.

The lymphatic system is an integral part of the circulatory system that is also a heterogeneous biofluid (Aspelund et al., 2016); its primary functions are to regulate fluid levels in the body, dispose of waste and toxins in tissue, and transport white blood cells to venous domains. The lymphatic system consists of lymph nodes, lymph vessels, and the spleen, which acts as a filter for blood. While the difficulties in lymph sample collection for viscosity measurement have been previously reported, microrheology overcomes these challenges due to its small sample requirement (Kassis et al., 2016). VPT has been used to investigate the effect of high lipid levels on lymphatic pump functionality (Kassis et al., 2016). In a rat model, the authors found that immediately after high lipid uptake, lymph viscosity increased by at least 50%, which led to an increase of the shear stress on lymph vessel walls. This information can quantify lymph vessel function and its relationship to diet imbalances and obesity.

Passive microrheology has been used to characterize the mechanical properties of cardiac tissue (Michaelson et al., 2012; Michaelson et al., 2014). Ma et al. used VPT and bulk rheology to characterize fibrin clots upon the infection by *S. epidermidis*. Assited by confocal imaging, the infected fibrin clots were observed to display increasing heterogeneity and viscoelasticity over time (Ma et al., 2017). To find the specific causes of diabetic cardiomyopathy, Kassis et al. investigated the biomechanical, contractile, and hypertrophic properties of cardiac muscles *via* VPT, DLS, and AFM. They found that the rigidity of cardiac muscles is correlated to blood sugar and fat imbalances. However, it was suggested that active microrheology may be more suited for investigating the relationship between the individual cardiac cell stiffness and overall heart muscle rheology.

Erythrocyte aggregation that forms at low shear rates has many clinical implications on blood disorders and tissue perfusion (Baskurt and Meiselman, 2013). The interconnections between the aggregation/deformation of red blood cells, the microscale viscosity of blood, and the pathology of many blood disorders are still not entirely understood. Microrheology facilitates the understanding and the development of diagnosis and treatment techniques of hematological problems. DLS has been used to assess blood aggregation (Priezzhev et al., 1999). However, this technique is inadequate in characterizing different types of erythrocyte aggregates, measuring the interaction forces within aggregates, or accounting for non-aggregated cells, leaving a knowledge gap in blood rheology and related pathologies. While passive microrheology has been extensively used to characterize blood and biomimetic analogs (Lévy et al., 2011), recent studies have utilized active microrheological methods to measure the biomechanical properties of red blood cell membranes (Liu et al., 2009; Robert et al., 2010) and cardiomyocyte tissue (Taylor et al., 2013).

**FIGURE 6:**
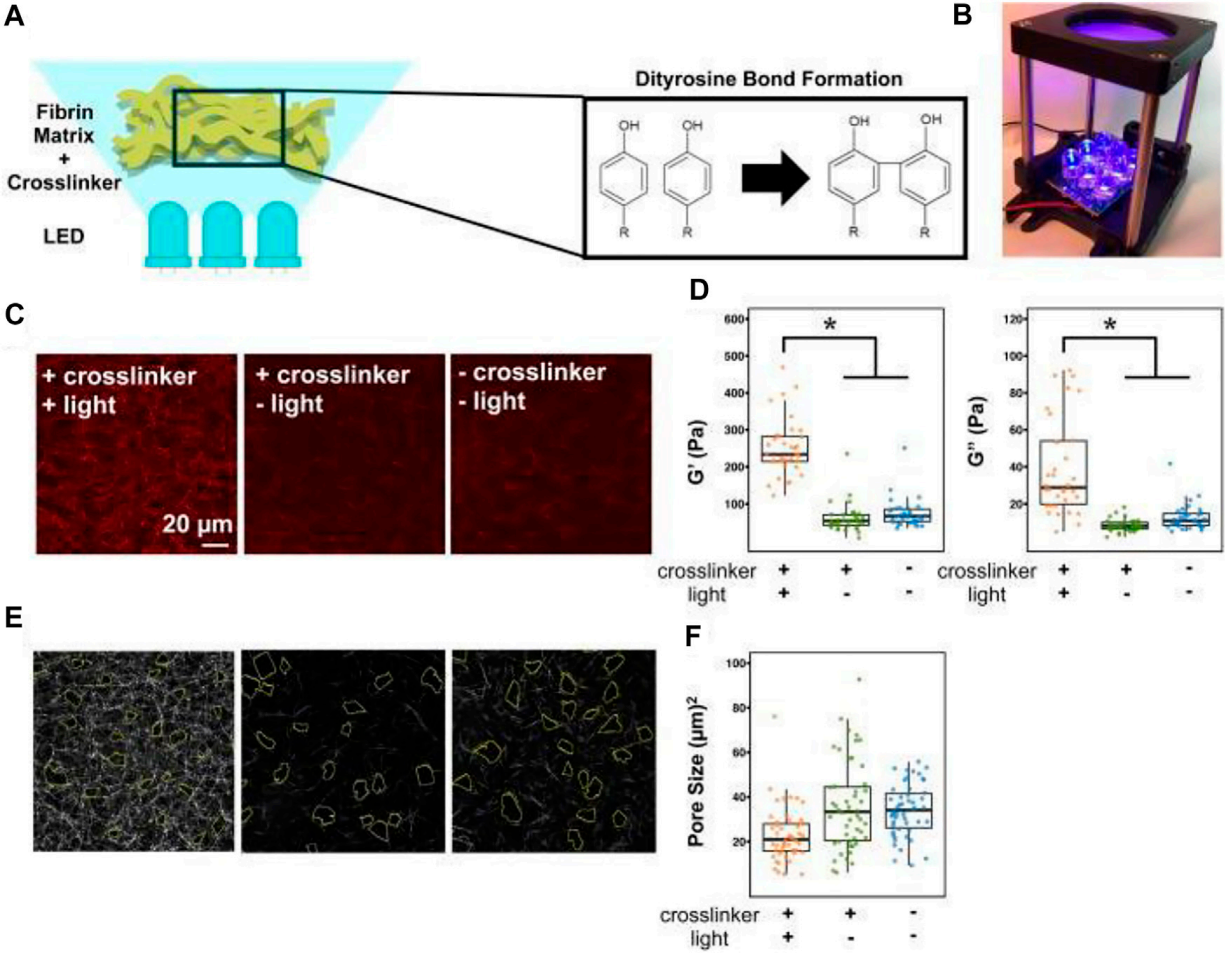
Viscoelastic and pore size characterization of fibrin gels. **(A)** Experimental schematic and **(B)** setup of photo-crosslinking process using 465 nm blue light. **(C)** Backscatter micrographs of 2 mg/ml fibrin gels at three different conditions. Scale bar is 20 microns. **(D)** Active microrheology scatterplots of measured storage (G′) and loss (G″) moduli using given conditions. The asterisk indicates *p* < 0.05 using the Mann-Whitney test with Bonferroni correction. **(E)** Micrograph with corresponding pore sizes outlined and **(F)** pore size scatterplots of the fibrin gels. Figure reproduced from Hsieh, Jessica Y., et al. “Matrix crosslinking enhances macrophage adhesion, migration, and inflammatory activation,” APL Bioengineering 3, 016103 (2019) https://doi.org/10.1063/1.5067301, with the permission of AIP Publishing.

OT has been used to measure the force interaction between the linear erythrocyte aggregates at the piconewton scale, providing researchers with insight into the development of the therapies that hinder erythrocyte aggregation and treat blood hyperviscosity (Maklygin et al., 2012). OT has also been used to characterize shear moduli of RBCs under different osmotic conditions as a stretching force being applied. The shear moduli were determined to be 10.5 ± 2.7, 17.3 ± 2.0, and 32.0 ± 4.0 μN/m for RBCs in hypotonic, isotonic, and hypertonic solutions, respectively (Youhua Tan et al., 2010). These results may potentially aid in identifying diseased cells and irregular plasma osmolality. Another application of OT on RBCs reported one particle and two-particle microrheology on red blood cells under an external stretching force. When combining OT with Raman spectroscopy, Raj et al. found that the nonlinear mechanical response of RBCs was caused by structural reorganization in the membrane-cytoskeleton system (Raj et al., 2013). Macrophages in blood and their relationship to the mechanical properties of the crosslinked fibrin of the ECM have recently been investigated using OT (Hsieh et al., 2019) (Figure 6). The positive relationship between ECM rigidity/density and macrophage spreading, adhesion, mobility, and inflammatory activation that researchers found using active microrheology provides information about the inflammation’s contribution to macrophage behavior during wound healing. Microrheology has also been used to evaluate the connections between lifestyle or environmental variables, blood aggregation, and resulting adverse symptoms that may negatively affect a patient’s life (Vorobyeva, 2017). *See* Table 5 for microrheological measurements in the circulatory system.

**TABLE 5:** Microrheological properties of mammalian cells and tissue of the circulatory system measured by various microrheological techniques.

Cell/tissue	Storage modulus (G′)	Loss modulus (G″)	Complex modulus (|G*|)	Scaling of storage modulus	Scaling of loss modulus	Scaling of complex modulus	Creep compliance (*J*)	Viscosity (η)	Pore size (ξ)	Reference
Cytoplasm of human umbilical vein endothelial cells	∼60 dyn/cm^2^ (1 Hz) VPT	∼11 dyn/cm^2^ (1 Hz) VPT								Fraley et al. (2011)
Non-crosslinked bovine fibrin gel	74.26 ± 39.5 Pa OT	∼10 Pa OT							35μm^2^	Hsieh et al. (2019)
Ruthenium photo-crosslinked bovine fibrin gel	251.76 ± 77.7 Pa OT	∼30 Pa OT							20μm^2^	
Whole human blood	∼0.02 Pa (10 Hz) VPT	0.04 Pa (10 Hz) VPT								Campo-Deaño et al. (2013)
Blood analogue: PAA (34 ppm)/HA (17 ppm)/Sucrose (35%)	∼0.01 Pa (10 Hz) VPT	∼0.05 Pa (10 Hz) VPT								
Blood analogue: XG (100 ppm)/DMSO (52%)	∼0.02 Pa (10 Hz) VPT	∼0.05 Pa (10 Hz) VPT								
Porcine cardiac thin myofilaments solution (3.66 μM) with Ca^+^				${\text{G}}^{\prime }∼\;\omega^{\frac{3}{4}}$ VPT	${\text{G}}^{\prime \prime }∼\;\omega^{\frac{7}{8}}$ VPT					Tassieri et al. (2008)
Porcine cardiac thin myofilaments solution (26 μM) with Ca^+^				${\text{G}}^{\prime }∼\;\omega^{\frac{1}{4}}$ VPT	${\text{G}}^{\prime }∼\;\omega^{\frac{3}{4}}$ VPT					
Porcine cardiac thin myofilaments solution (1–7 μM) with Ca^+^				${\text{G}}^{\prime }∼\;c^{0.9\pm 0.1}$ VPT	${\text{G}}^{\prime }∼\;c^{0.9\pm 0.1}$ VPT					

In the circulatory system, passive microrheological methods have been found to be inadequate in describing the mechanical properties of tissue and cells, especially for cardiac tissue and erythrocyte cells and aggregates. Active microrheological methods, specifically OT, has shown its advantages in this system in connecting the erythrocyte aggregating behavior with blood disorders and measuring the interaction force between these cells, as well as the interaction between red blood cells and the ECM.

### Integumentary System

The human integumentary system is comprised of skin, breast tissue, fatty subcutaneous tissue, and various accessory organs such as hair, nails, sweat glands, and sebaceous glands. The skin is the body’s largest organ and primarily serves as a chemical/mechanical protection barrier, a homeostasis regulator, a production factory of signaling molecules and structural proteins, and a sensory domain (Chuong et al., 2002). Hair and hair follicles are crucial for sensing, thermoregulation, and protection. Subcutaneous tissue, located beneath the dermis and consisting of adipose cells, serves as an insulator, shock absorber, energy stockpile, and hormone regulator (Kershaw and Flier, 2004). The subcutaneous tissue also encompasses the breast, a fatty superficial organ whose glands produce milk for offspring, which can also be classified as an accessory female reproductive organ.

Skin and gland disease, growth, and aging are interconnected with the interactions between cells and the ECM. These relationships are critical in tumor progression and metastasis. These diseases have been examined using passive and active microrheology in the tissue grafts and 3D epithelial organoid models. Microrheologcial assessments provide information on the mechanotransduction of skin and breast cancer that facilitates the development of diagnostic tools and treatments.

Like many other cancers in the human body, integumentary cancer behavior/signaling, and ECM stiffness have been shown to be related, but the mechanisms are under investigation. Although previous studies mostly used bulk rheology to characterize tumor ECM (Wu et al., 2018b), the viscoelastic properties of cellular environments on the cellular level have been shown to differ significantly from the bulk measurements. For example, data generated by OT uncovered that dermal fibroblasts and breast cancer cells unevenly stiffen their hydrogel ECM on the single-cell level, but induce an overall stiffening effect on the bulk scale (Jagiello et al., 2020). This spatial gradient and anisotropy in the cellular microenvironment are not assessable by conventional rheometry. DLS has been used to investigate breast cancer mechanics (Arosio et al., 2008). Combining DLS and confocal microscopy, two breast cancer models were investigated and visualized: tissue remodeling *via* stromal cell contracting and breast cancer spheroid invasion (Krajina et al., 2021a). The results suggested that simultaneous ECM fluidization and stiffening both play an important role in the breast cancer cells’ migration throughout the tissue. Interestingly, besides synthesized tracer particles, intracellular mitochondria have also been used as tracers in recent microrheological research in breast cancer models (Mak et al., 2014). By combining mitochondria-tracking, microfluidics, and Brownian motion simulations, Staunton et al. showed that the intracellular microrheological properties of metastatic MDA-MB-231(human breast epithelial carcinoma) cells in 3D culture are more elastic than those of the cells grown in 2D environment. These results agree with literature findings (Staunton et al., 2019). Furthermore, the effects of chemotherapy (i.e., paclitaxel) on breast cancer cells are also qualitatively and quantitatively reflected in the intracellular mechanical properties, as shown in a 2013 study using VPT on drug-treated 2D breast cancer cell cultures (El Kaffas et al., 2013). The results showed that after chemotherapy exposure of 24 h, G′ and G″ reported at 1 rad/s of the cancerous cytoplasm were found to increase by 191.3 Pa (>8000 folds) and 9 Pa (∼9 folds), respectively (Table 6). The mechanical change of cancer cells in response to the treatment can potentially be utilized to monitor and evaluate the efficacy of chemotherapy.

**TABLE 6:** Microrheological properties of mammalian cells and tissue of the integumentary system measured by various microrheological techniques.

Cell/tissue	Storage modulus (G′)	Loss modulus (G″)	Complex modulus (|G*|)	Scaling of storage modulus	Scaling of loss modulus	Scaling of complex modulus	Creep compliance (*J*)	Viscosity (η)	Pore size (ξ)	Reference
Nonmalignant MCF10 embedded in laminin-rich ECM (measured at ≥ 50 μm)			∼20 Pa (100 Hz) OT					∼0.3 Pa s (100 Hz) OT		Staunton et al. (2019)
Nonmalignant MCF10 embedded in laminin-rich ECM (measured at ≤ 10 μm)			∼35 Pa (100 Hz) OT					∼0.3 Pa s (100 Hz) OT		
Intracellular measurement of nonmalignant MCF10			∼45 Pa (100 Hz) OT					∼0.4 Pa s (100 Hz) OT		
Malignant MCF10-CA1 embedded in laminin-rich ECM (measured at ≥ 50 μm)			∼12 Pa (100 Hz) OT					∼0.5 Pa s (100 Hz) OT		
Malignant MCF10-CA1 embedded in laminin-rich ECM (measured at ≤ 10 μm)			∼50 Pa (100 Hz) OT					∼0.5 Pa s (100 Hz) OT		
Intracellular measurement of malignant MCF10-CA1			∼100 Pa (100 Hz) OT					∼0.5 Pa s (100 Hz) OT		
Cytoplasm of MCF-7 before treatment	0.022 Pa (1 rad/s) VPT	1.1 Pa (1 rad/s) 1.2 VPT								El Kaffas et al. (2013)
Cytoplasm of MCF-7 after 24 h of paclitaxol exposure	191.3 Pa (1 rad/s) VPT	10.1 Pa (1 rad/s) VPT								
Encapsulated human mammary fibroblasts cultured in col/rBM (day 6)	180 Pa (1 rad/s) DLS	30 Pa (1 rad/s) DLS								Krajina et al. (2021b)
Encapsulated human mammary fibroblasts cultured in col/rBM (day 6 + depolymerization by latrunculin A)	90 Pa (1 rad/s) DLS	12 Pa (1 rad/s) DLS							
MCF-7 cells	4.5 ± 0.4 Pa (1 Hz) VPT	10.1 ± 0.9 Pa (1 Hz) VPT								Wu et al. (2018b)
B16-F10 melanoma tumors on mouse *ex vivo* (2 nm probes)						|G*| ∼ ω^0.70^				Staunton et al. (2016)
B16-F10 melanoma tumors on mouse *ex vivo* (5 nm probes)						|G*| ∼ ω^0.63^				
B16-F10 melanoma tumors on mouse *ex vivo* (10 nm probes)						|G*| ∼ ω^0.57^				
B16-F10 melanoma tumors on mouse *ex vivo* (20 nm probes)						|G*| ∼ ω^0.52^				
B16-F10 melanoma tumors on mouse *ex vivo* (2, 5, 10, 20 nm probes)	4–20 Pa (10 Hz) OT	5–20 Pa (10 Hz) OT								
B16F10 Melanoma tumor on mouse *ex vivo*	∼30 Pa (10 Hz) OT	∼80 Pa (10 Hz) OT								
MDA-MB-231 Metastatic Breast tumor on mouse *ex vivo*	∼140 Pa (10 Hz) OT	∼200 Pa (10 Hz) OT								

**FIGURE 7:**
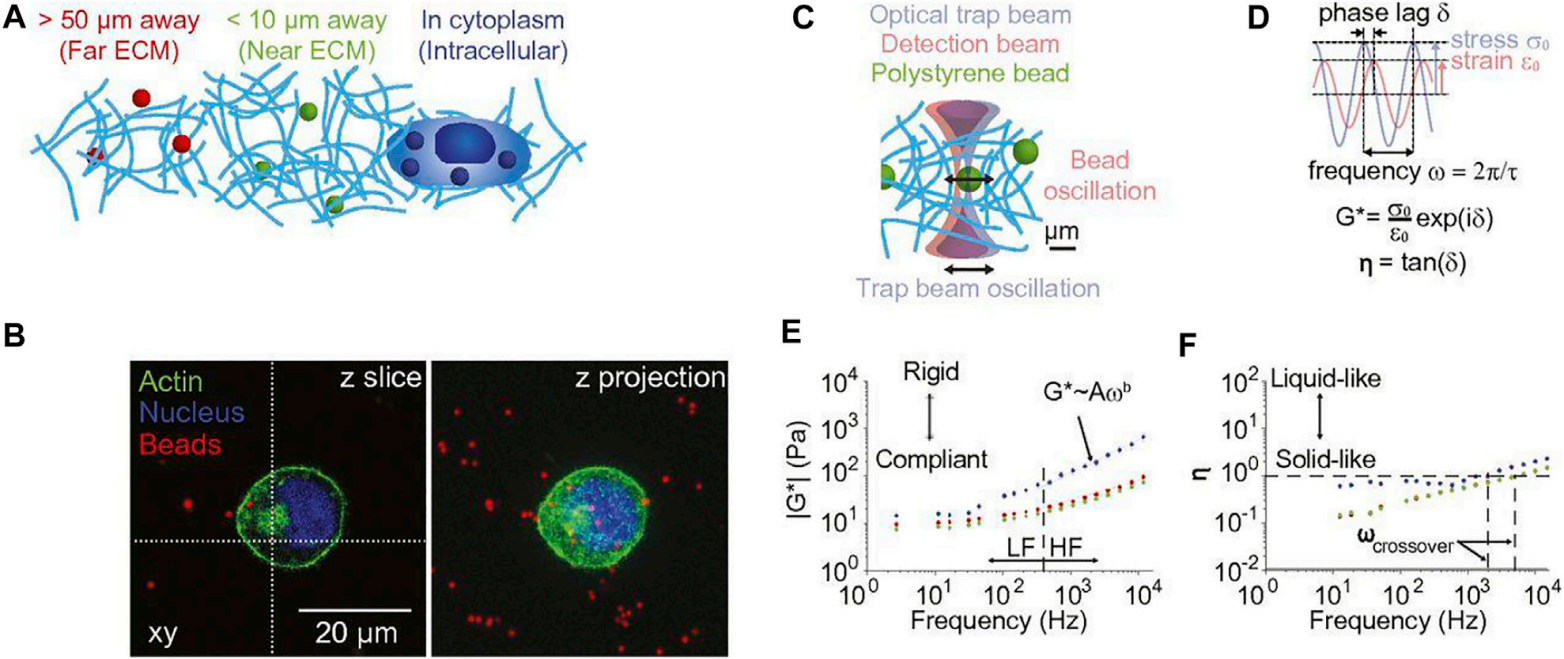
Active microrheology using optical tweezers inside cells and in the extracellular matrix (ECM) near and far from the cell. **(A)** Illustration of one micron particles probing inside the cells and in the ECM. **(B)** Confocal images showing the distribution of particles (red). **(C)** Illustration of oscillating a probe by the optical trap in the medium. **(D)** Dynamic parameters obtained from the optical trapping measurement. **(E)** Frequency dependent complex modulus shows power law dependence in the high frequency regime. **(F)** Hysteresivity (η = G″/G′) shows the liquid-like or solid-like dominating property of the material. Figure from J. R. Staunton, W. Y. So, C. D. Paul, and K. Tanner, “High-frequency microrheology in 3D reveals mismatch between cytoskeletal and extracellular matrix mechanics,” Proceedings of the National Academy of Sciences, vol. 116, no. 29, pp. 14448–14455, 2019 with permission from PNAS.

Although passive microrheology has been implemented to examine the viscoelastic properties of integumentary cancers, active microrheology has recently been favored to study tumor mechanics at the microscopic scale. OT has been utilized to determine the relationship between molecular regulation of the breast cancer invasion process and the effects on the mechanical properties of cell cytoplasm within 3D tumor models (Han et al., 2020). Han et al. used OT to quantify the stiffness of breast cancer cells as a function of tumor depth. They found that cells in the core of the cancer organoids were the most rigid, and the cells on the peripheral of the cancer models were softer, larger, and more invasive. This heterogeneous pattern of tumor uncovers its metastasis mechanism (Table 6) (Han et al., 2020). Differences between the mechanical properties of breast epithelial cells and ECM have also recently been measured using OT. Although it has been reported that cancer cells soften during the malignant procession, Staunton et al. found that the mechanical properties of normal and cancer cells are highly context-dependent (Staunton et al., 2019). These cells adjust their mechanical properties and the ECM over time. As a result, the intracellular and extracellular mechanical properties were similar in healthy non-malignant breast tissue, while a mismatch between the intracellular and extracellular stiffness of human breast epithelial carcinoma (cell line MCF10-CA1) cells was observed (Figure 7). However, establishing the correlation between the change of mechanical properties and the chemical cues requires further interrogation.

Melanoma is a common type of skin cancer that originates in the melanocytes and is often caused and exacerbated by UV-ray damage. Melanoma has been investigated with both active and passive microrheology. OT and bulk rheology were used to measure the aberrant ECM mimicked by type I collagen hydrogels, showing agreement in their overlapping frequency range. Additionally, *ex vivo* murine melanoma tumor and human breast tumor samples were also probed with OT. The resulting |G*| of both tumor samples fall in the range of 5–1,000 Pa measured within 3–15,000 Hz (Table 6) (Staunton et al., 2016). In addition to active microrheology, passive microrheology *via* VPT has been employed recently to investigate the mechanics of melanoma cancer cells. A report tracked the motion of melanoma mitochondria during three growth phases to explore the connections between tumor cell deformability, mobility, and microtubule structure (Higgins et al., 2020). By relating the MSD of intracellular mitochondria to intracellular fluidity, researchers found increased fluidity associated with elevated content of actin and tubulin in cells, which may be exploited to serve as an indicator of cell migration (Higgins et al., 2020).

### Urinary System

The human urinary system consists of the upper urinary tract and the lower urinary tract. The upper urinary tract (UUT) includes the kidneys and the ureters. It filters waste from the blood, regulates body electrolyte levels, and transports urine to be stored in the bladder. The lower urinary tract (LUT) consists of the bladder and urethra; the LUT’s function is to store urine and transport it out of the body. Since nephrons are the driving force behind salt balance and transport within the kidney, understanding the diffusion and fluid dynamics within renal tissue is crucial for developing treatments for urinary tract infections and disorders.

Renal cell biology at the intracellular level facilitates the understanding of blood filtration mechanisms and regulation. Kidney cells are relatively easy to harvest and culture, thus are widely used to explore the intracellular mechanics. In a recent report, single-particle tracking microrheology was used to understand the effect of lysosome size on its transport inside monkey kidney epithelial cells (Bandyopadhyay et al., 2014). Using the lysosome itself as a spherical probe particle with a size range of 100 nm – 5 μm (lysosome enlarged by osmotic swelling), Surendran et al. found that intracellular diffusive transport is reversely correlated with lysosome size. In contrast, the size of an enlarged lysosome showed no effect on its active transport. Studying organelles is particularly important in the kidney; lysosome abnormalities have been linked to kidney diseases, such as Fabry’s disease, renal failure, and cystinosis caused by abnormities in transporting proteins during glomerular filtration (Surendran et al., 2014). Other passive tracking techniques have also been developed following the same microrheological theories and parameters. For example, laser tracking microrheology (LTM) is a technique established in 2000 (Yamada et al., 2000) as a non-invasive modality for examining intracellular mechanics *via* image tracking of the Brownian motion of endogenous structures in the cytoplasm. Spherical, refractile granules made of lipids within monkey kidney fibroblast cells (cell line COS-7) were fluorescently stained and tracked using laser optics at a small length and time scale (Table 7. Microrheological properties of mammalian cells and bodily fluids of the urinary and reproductive systems measured by various microrheological techniques) (Yamada et al., 2000). Cytoplasmic mechanical measurements of kidney cells provide helpful information about the changes in intracellular behavior in response to cargo and fluid transport within nephrons.

**TABLE 7:** Microrheological properties of mammalian cells and bodily fluids of the urinary and reproductive systems measured by various microrheological techniques.

Cell/tissue	Storage modulus (G′)	Loss modulus (G″)	Complex modulus (|G*|)	Scaling of storage modulus	Scaling of loss modulus	Scaling of complex modulus	Creep compliance (*J*)	Viscosity (η)	Pore size (ξ)	Reference
COS7 cytoplasm measured by perinuclear spherical lipid-storage granules			∼100 Pa (1 Hz) LTM^a^							Yamada et al. (2000)
COS7 cytoplasm measured by lamellar spherical lipid-storage granules			∼500 Pa (1 Hz) LTM^a^							
Primary ovarian cancer cell OV445							0.3 Pa^−1^ (Maximum) MT			Swaminathan et al. (2011)
Primary ovarian cancer cell OV207							3.1 Pa^−1^ (Maximum) MT			
Ovarian cancer cell HEY, SKOV3, and 429neo							∼0.9 Pa^−1^ (Maximum) MT			
Ovarian cancer cell 2008 and 420							∼0.5 Pa^−1^ (Maximum) MT			
Ovarian cancer cell DOV13							∼0.3 Pa^−1^ (Maximum) MT			
Ovarian cancer cell DOV13							∼0.1 Pa^−1^ (Maximum) MT			
Cytosol of HeLa cells				G’ ∼ ω^0.5^ OT	G’ ∼ ω^0.75^ OT					Nishizawa et al. (2017)
HeLa cells at anaphase	5 Pa (10 Hz) VPT	10 Pa (10 Hz) VPT								Chen et al. (2014)
Human cervicovaginal mucus, pH 1–2									430 ± 70 nm VPT	Wang et al. (2013)
Human cervicovaginal mucus, pH 4									370 ± 60 nm, 310 ± 50 nm VPT	
Human cervicovaginal mucus, pH 6–7									360 ± 60 nm VPT	
Human cervicovaginal mucus, pH 8–9									210 ± nm VPT	

a Laser tracking microrheology.

Single-particle tracking has also been used to investigate mechanisms of diffusion obstruction in the cellular membrane of kidney cells (Weigel et al., 2011), specifically the role of the voltage-gated potassium channel Kv2.1 and actin cytoskeleton filaments on trans-membrane transport. The experimental data was validated by the diffusion obstruction model simulated using the Monte Carlo method (Weigel et al., 2012). Other studies using single-particle tracking microrheology have looked at the change of diffusion coefficient in the plasma membrane induced by the regulatory mechanism of aquaporin-5 (AQP5) (Koffman et al., 2015). Results showed that the diffusion of AQP5 was highly regulated by cAMP, PKA, and T259 phosphorylation.

The outer epithelium of the bladder is an apical surface covered by a mucus membrane that protects the organ from microbes and pathogens. However, the mucus layer also hinders drug penetration into the organ. Successful penetration to the underlying bladder tissue *via* intravesical drug delivery cannot be evaluated using bulk rheology but microrheology. In a recent report, VPT was performed to measure the diffusion efficiency of polystyrene (PS) nanoparticles with different surfaces in *ex vivo* porcine bladder mucosa. As surface PEG-ylation has been widely used to enhance particle mobility in mucus, this report showed carboxylated PS nanoparticles coated with hydrophilic polydopamine (PDA) have similar mobility as the PEG-PS counterparts. Besides enhancing mucopenetration, this surface alteration by PDA of particles also allows for photothermal therapy on the bladder tissue. (Poinard et al., 2019).

In addition to characterizing urinary tissue and mucus, microrheology has also been employed to aid in the development of biomaterials and tissue-printing techniques in the urinary system. Acellular ECM biological scaffolds have been extensively used in tissue engineering for tissue reconstruction and modeling for their inherent biocompatibility. Bladder acellular matrix (BAM) is derived from the bladder and contains growth factors that facilitate bladder tissue regeneration. Thus BAM hydrogel scaffolds have recently been developed as a treatment for urinary incontinency and bladder injury repair. Jiang et al. used DWS to characterize the gelation time of BAM hydrogels, which were as short as 3.95 ± 0.07 min (Jiang et al., 2017). The Solid-Liquid Balance (SLB) = 0.5 served as the criteria to identify the gel point. The SLB here is the diffusive exponent (*α*) of the MSD. The relation $\langle \Delta r^{2}\left(t\right)\rangle ∼t^{0.5}$ has been used in microrheology to determine the gel point based on the Rouse model (Savin and Doyle, 2007), as discussed in Section 2.1.4.

Various experimental strategies have been employed for microrheological measurements in the urinary system, but passive methods are dominantly used. Passive tracking is particularly suitable for studying the filtration mechanism and drug particles’ penetration efficiency in this system. Intracellular organelles such as lysosomes and spherical cytoplasmic structures were used as the probes to characterize intracellular mechanical properties. The accessibility and vitality of kidney cells make the urinary system an excellent platform for microrheological exploration and advancement.

### Reproductive System

The female reproductive system includes the internal and external structures that conceive and nurture offspring. The internal organ system consists of the vaginal canal, cervix, uterus, fallopian tubes, and ovaries. Ovarian epithelial cells secrete oviduct fluid and provide a suitable environment for fertilization, oocyte transport down the fallopian tubes, and sperm swimming (Miller, 2015). Vaginal mucus is secreted by the glands near the cervix and vaginal opening; it functions as a protective barrier as well as a pH and microbiome regulator (Wang et al., 2013; Chee et al., 2020).

A 2010 study performed VPT to evaluate the pore size of healthy human cervicovaginal mucus (CVM). Four different sizes of PEG-coated particles ranging from 100 nm to 1 μm were used to characterize the pore size of the CVM. As a result, the average pore size of healthy CVM was determined to be 340 ± 70 nm. This pore size was much larger than the prediction and the size of most mammalian viruses. However, the penetration of the herpes simplex virus (HSV) with a size of 180 nm in the CVM was significantly obstructed by the network (20,000 folds slower than HSV diffusing in water) (Lai et al., 2010). Lai et al. hypothesized that vaginal mucus gel protects the body against infection through adhesion mechanisms, trapping viruses and microbes, rather than steric obstruction (Lai et al., 2010). Another report used VPT to examine the interaction between drug-loaded polycaprolactone (PCL) microbicidal nanocarriers of various surfaces with simulated vaginal fluid (SVF) infused with mucin molecules (das Neves et al., 2012). Das Neves et al. used both bulk rheology and VPT to characterize the mechanical properties of SVF at different pH levels, modeling the vaginal mucus microenvironment at normal conditions, during infection, and in the presence of semen. Like reported in literature, Das Neves et al. found that negatively charged nanoparticles exhibited higher mobility than positively charged counterparts and displayed the potential to be the surface of drug delivery vehicles (das Neves et al., 2012). In a later study examining fresh cervicovaginal fluid, both VPT *via* PEG-coated fluorescent particles and bulk rheology were employed to explore the effects of pH on the microstructure of native mucus. By extrapolating pore size from particle motion, Wang et al. found that increasing the pH of the vaginal mucus reduced the pore size from 370 ± 60 nm (pH ∼ 4) to 210 ± 40 nm (pH = 8–9), but the interaction between mucus and particles were not substantially altered; however, decreasing pH values induced pore size to increase from 310 ± 50 nm (pH ∼ 4) to 430 ± 70 nm (pH = 1–2). Its overall structure was stable at mid-range pH values, suggesting that easily-accessible vaginal mucus is a suitable model for further microrheological study as an *ex vivo* model for other neutral physiological mucus (Wang et al., 2013).

Understanding the microrheology of female reproductive fluids and tissues not only aid in improving treatments for infertility and vaginal infection, but also provides insight into gynecological cancers. The most used human cell line in biomedical research are HeLa cells, an immortal culture of cervical cancer (Masters, 2002). Using HeLa cells, the mechanical properties of gynecological tumors have been related to their progression and metastasis. The heterogeneity of HeLa cells has been investigated using a variety of active and passive rheological methods. Swaminathan et al. used MT to measure the mechanical properties of ovarian cancer cells and demonstrated that cancer cell stiffness and invasiveness show an inverse correlation, which is likely attributed to higher deformability that promotes tumor metastasis. These mechanical phenotypes may be utilized as a marker for ovarian tumor progression (Swaminathan et al., 2011). In another report, researchers sought to use magnetic rotational microrheology to measure the intracellular viscosities of HeLa cells. The magnetic probes were formed by labeling the intracellular endosomes with magnetic particles and aligning them to create chains within the cytoplasm of the HeLa cells. Then, the intracellular microrheological properties were quantified upon the application of a rotational magnetic field to the magnetic endosome chains. As a result, a high degree of heterogeneity of the cell interior was observed (Wilhelm et al., 2003a).

Passive microrheology has also been used to study the mechanical properties of cervical cancer cells, specifically the relationship between HeLa cell cytoskeleton structure and intracellular viscosity (Chen et al., 2014). Probes larger than the cytoskeleton network pore size (100 nm) was used to target and measure the changes in the viscoelasticity of the cytoskeleton during cell division. Results showed that both G′ and G″ increased as HeLa cell mitosis moved from metaphase to anaphase and remained constant from anaphase to telophase. These results have broad implications on the effects of cytoskeleton structure on tumor propagation and growth (Chen et al., 2014). Recently, passive and active microrheology have also been used simultaneously to examine the mechanics of epithelial-like HeLa cells. In 2017, Nishizawa et al. used both OT technology and laser interferometry to characterize the intracellular microrheological properties in the presence of cytoplasmic fluctuations. It was found that the living cytoplasm inside of epithelial-like HeLa cells displayed glass-like behavior with $G^{\text{*}}\propto {\left(-i\omega \right)}^{\frac{1}{2}}$ (Nishizawa et al., 2017). *See* Table 7. Microrheological properties of mammalian cells and bodily fluids of the urinary and reproductive systems measured by various microrheological techniques for the microrheological measurements using different techniques in the reproductive system.

**FIGURE 8:**
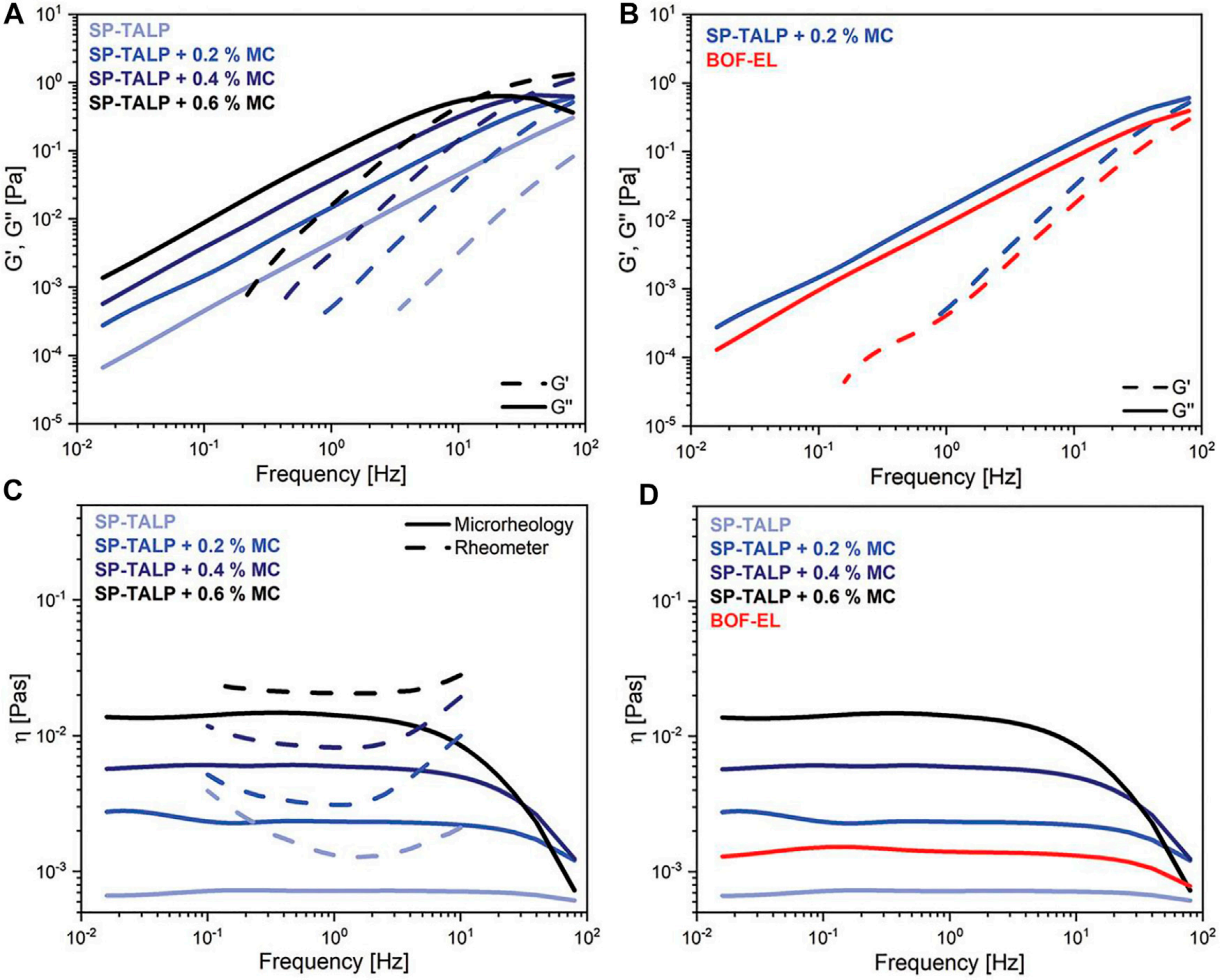
Video particle tracking (VPT) microrheology and bulk rheology characterization of bovine oviduct fluid (BOF) and viscoelastic methycellulose (MC)- enriched analogues. **(A)** Storage modulus G′ (dashed line) and loss modulus G″ (solid line) as a function of frequency of MC-enriched SP-TALP media obtained from VPT at physiological temperature. **(B)** Storage and loss modulus of 0.2% MC and BOF-EL measured from VPT. **(C)** Microrheology measurements (solid line) overlaid by rotational rheometer measurements (dashed line) at physiological temperature. **(D)** Corresponding viscosities calculated from VPT data. Figure from Striggow, Friedrich, et al. “Sperm‐Driven Micromotors Moving in Oviduct Fluid and Viscoelastic Media.” *Small*, vol. 16, no. 24, 2020, p. 2000213., doi:10.1002/smll.202000213. 00213., doi:10.1002/smll.202000213. Original work is available for use under the Creative Commons CC BY license.

Sperm, or the male reproductive gamete, are motile, uniflagellate cells that utilize the oviduct fluid for optimal swimming speed. Recent developments in microrobotics have employed sperm cells into biohybrid “spermbots,” with potential applications for fertilization *in vivo*. In a 2020 study, VPT was utilized to characterize the microrheological properties of bovine oviduct fluid (BOF) to design artificial oviduct fluid since BOF samples are difficult to harvest. As a result, it was found that the viscosity of BOF was two to three times higher than water and a commonly used sperm swimming media SP-TALP, while SP-TALP with 0.2% methylcellulose (MC) addition showed rheological properties that match BOF and can serve as a test substitute of BOF (Figure 8) (Striggow et al., 2020). With this test media, the authors analyzed the motion of free sperm cells and proposed a design of spermbot consisting of a magnetic streamline cap propelled by attached sperm flagella as well as an external static magnetic field (Striggow et al., 2020).

In the female reproductive system, VPT has been favored for microheological measurements in the last decade, especially for investigating vaginal mucus. Cancerous vaginal tissue displays higher mechanical properties, and active microrheological characterization will provide even more insight into its rheological behavior. VPT is particularly suitable for evaluating virus penetration, drug delivery, and sperm swimming in the vaginal fluids. Therefore, this technique shows potential to assist in the diagnosis of vaginal disease associated with changes in the micheorheological properties of vaginal fluid. VPT is also a useful testing approach for drug delivery research and facilitated sperm swimming in the vaginal environment.

## Summary and Future Prospects

Microrheology is increasingly being used in biomedical investigations to obtain measurements with spatiotemporal information that reveals the nanoscale heterogeneity of biological environments. Additionally, microrheological techniques require minimal sample volume can often operate non-invasively; microrheology has great potential to serve as complementary medical diagnostic methods, especially when native biofluids or tissue samples are difficult to collect, such as arthritis joint, lymph, and intracranial cerebrospinal fluid. Micro/nanoparticles can be phagocytosed by cells and intracellular organelles such as mitochondria and lysosomes. Therefore, intracellular mechanical properties and activities can be evaluated by the endocytosed probe particles or directly by the intracellular organelles, providing more non-invasive options. The wide selection of size, surface, and body material of micro/nanoparticles, as well as the selection of probing technique, provide microrheology with the versatility to meet the need of experiment and to acquire desired results, such as diffusion coefficient of drug delivery systems or virus penetrating mucus media, viscoelasticity of cells and ECM as a complementary diagnostic method describing disease progression, and intracellular activities during mitosis. Tables 2–7 list rheological properties of mammalian cells and tissue that were evaluated using microrheological techniques in the previously discussed articles. Future innovations in the probe particles include advanced surface functionalization that allows them to adapt to more environments in biological systems. For example, probe particles phagocytized by immune cells *in vivo* can be used to explore and monitor autoimmune disorders, of which the causes are still mostly unclear. Another application could be using functionalized micro/nanoparticles to understand the selectivity mechanism of the blood-brain barrier and related disorders.

Furthermore, microrheological properties can be used to determine the resemblance in the microrheological properties between synthetic and natural biomaterials and further improve the design of artificial biofluids and tissues. Great efforts have been made to develop biomaterials used for tissue repair and regeneration that attempt to modulate the human body’s immune response after transplantation and aid in the healing process (Karkanitsa et al., 2021). In addition, a connection is expected to be made between rheological behavior and tissue histology micrographs (Curtis et al., 2019), paving the way for faster diagnostic procedures. Differential dynamic microscopy (DDM) is a passive microrheology technique that combines MPT with light scattering (Bayles et al., 2016, Edera et al., 2017). DDM does not require user input. Taking this advantage, Martineau et al. built an automated system with DDM and machine learning algorithms to specify hydrogel formulation (Martineau et al., 2022). One can envision utilizing machine-learning algorithms taking microrheological probe trajectories of *ex vivo* or *in vivo* tissue as an input, and controlling a 3D bioprinter to replicate organ tissue for patient transplant, disease research, and natural bioproduct production, potentially minimizing the need for organ donations (Abouna, 2008).

*In vivo* microrheology also opens up doors in developmental biology and embryology (Wirtz, 2009). Particle tracking microrheology has recently been used to explore the evolution of the mechanical properties of zebrafish embryo yolk cells during epiboly, a cell movement during early embryonic development (Marsal et al., 2017). This report highlights the non-invasiveness of microrheology in investigating fragile and soft tissue under development. In the future, microrheology may be employed to study the physical changes during mammalian embryonic development at the microscale to aid in the investigation of the causes and symptoms of pregnancy complications.

In the future, we envision both active and passive techniques to be used *in vivo* in the clinical setting to investigate and monitor tissue in afflicted patients in a non-invasive, cost-effective, and accurate manner. Recently, magnetic microprobes have been utilized to provide intracellular mechanical measurements of individual organelles (Wang et al., 2019). Mechanical properties of the cell nucleus and cytoskeleton have been linked to a wide variety of diseases and cancers. Due to the subcellular scale and hard-to-reach location of the diagnostic structures inside cells, microrheology remains the most potential technique to probe these areas. Technologies incorporating microrheology may be developed to involve mechanical phenotype to explain the cause and progression or aid in the diagnosis of diseases (Mukherjee et al., 2021). One can envision using microrheological techniques to map the structure and mechanical properties of the brain in real-time on a patient-by-patient basis, enabling researchers to monitor neural changes in response to various stimuli and disease progression. Additionally, further understanding of the mechanical properties of brain tissue *in vivo* could unlock avenues to develop brain-like biomaterials; due to a lack of knowledge of brain tissue’s anisotropic and relatively soft structure, materials resembling this organ have yet to be successfully engineered (Axpe et al., 2020). Combining the knowledge of designing brain-like tissue with machine learning, one may expect future regenerative medicine using biomaterials tailored to individual brain structures developed by these techniques.

Recently, active particles in the form of micro and nanoscale motors and robots have shown great potential for future *in vivo* theranostics (Koleoso et al., 2020; Soto et al., 2020). The mechanics of fluidic microenvironments are critical for the development of these small scale wireless devices as propulsion dynamics are very different in Newtonian fluids versus viscoelastic solutions. For example, magnetically propelled helical nanostructures, with sizes comparable to natural polymer mesh networks, can achieve higher speeds in gels than in water (Schamel et al., 2014). Magnetic propelled helices themselves have been used as mobile microrheometers for Newtonian fluids, complex fluids, intracellular and extracellular environments (Ghosh et al., 2018; Dasgupta et al., 2020; Pal et al., 2020; Ghosh and Ghosh, 2021). Similarly, Janus silica particles have been shown to self-propel and display orientation fluctuation upon laser activation. The translational and rotational motion of these particles have been used to characterize the micromechanical properties of colloid glassy systems (Lozano et al., 2019). More recently, bio-hybrid micromotors which couple synthetic structures with biological components have drawn particular interest for their multifunctionality and biocompatibility (Schwarz et al., 2017; Quashie et al., 2022). For example, sperm have been incorporated into the design of micro swimmers to improve their mobility. Investigations have also explored the significant effects of viscoelasticity on microorganism motility, including sperm (Tung et al., 2017; Ishimoto et al., 2018). A recent review by Li et al. discussed how cilia and flagella propel cells differently in complex materials, heterogeneous environments, and under external flow (Li et al., 2021). We envision in the future that engineered microorganisms, bio-hybrid micromotors and nanorobots will be used as active probes for both microrheological characterization and therapeutic agents. For instance, next generation therapeutics may employ swarms of nanoscale swimmers, directed by self-propulsion or an external field, to a tumor site where these agents are then used for *in vivo* microrheological characterization, providing localized mechanical phenotyping for subsequent targeted drug delivery or precision surgery.

Unfortunately, translation from predominant *in vitro* and *ex vivo* test conditions to clinical operations is progressing slowly. A large number of trials should be performed before this translation to prove the safety and efficacy of all the techniques mentioned above. However, many fresh biological samples are hard to collect and preserve. More importantly, the stability and biocompatibility of these particles must be proved before translating experimental achievements to clinical operations. Biodegradable polymers have been extensively investigated and implemented in biological systems and show great potential in biomedical applications (Elmowafy et al., 2019; Idrees et al., 2020). Feridex (USA) is an MRI contrast agent containing iron oxide particles coated with dextran. This product showed biocompatibility and biodegradability and was previously approved by the FDA. The design of the dextran coating is intended to prevent particle coagulation; however, the product was later taken off the market because the dextran surface also hinders cell uptake of these particles (Markides et al., 2012; Reddy et al., 2012). Additionally, nondegradable probe particles should be retrieved after performing assigned tasks (Tersteeg et al., 2014; Lee et al., 2021); however, fewer reports have been found supporting successful retrievals of micro/nanoparticles.

Although there are challenges to overcome, microrheology is rapidly evolving in the biomedical field, providing great potential for understanding many biological processes at the molecular level and enabling possible diagnostic approaches. The versatility of microrheological techniques provides the field of nanomedicine with many possibilities. One can anticipate more applications of microrheology to be developed and increasingly used in biomedical fields, incorporating emerging techniques and theranostic approaches.
